# Electronic Health Interventions to Improve Adherence to Antiretroviral Therapy in People Living With HIV: Systematic Review and Meta-Analysis

**DOI:** 10.2196/14404

**Published:** 2019-10-16

**Authors:** Ziqi Wang, Yaxin Zhu, Liyuan Cui, Bo Qu

**Affiliations:** 1 School of Public Health China Medical University Shenyang China; 2 School of Medical Informatics China Medical University Shenyang China

**Keywords:** HIV, highly active antiretroviral therapy, medication adherence, eHealth

## Abstract

**Background:**

Electronic health (eHealth) is increasingly used for self-management and service delivery of HIV-related diseases. With the publication of studies increasingly focusing on antiretroviral therapy (ART) adherence, this makes it possible to quantitatively and systematically assess the effectiveness and feasibility of eHealth interventions.

**Objective:**

The purpose of this review was to explore the effectiveness of eHealth interventions on improving ART adherence in people living with HIV. The effects of different intervention characteristics, participant characteristics, and study characteristics were also assessed.

**Methods:**

We systematically searched MEDLINE (via PubMed), EMBASE, the Cochrane Central Register of Controlled Trials, and 3 conference abstract databases using search terms related to HIV, ART, adherence, and eHealth interventions. We independently screened the studies, extracted the data, and assessed the study quality and then compared the information in pairs. Articles published in English that used randomized controlled trials to assess eHealth interventions to improve ART adherence of people living with HIV were identified. We extracted the data including study characteristics, participant characteristics, intervention characteristics, and outcome measures. The Cochrane risk-of-bias tool was used to assess the risk of bias and study overall quality. Odds ratios, Cohen *d*, and their 95% CIs were estimated using random-effects models. We also performed multiple subgroup analyses and sensitivity analyses to define any sources of heterogeneity.

**Results:**

Among 3941 articles identified, a total of 19 studies (including 21 trials) met the inclusion criteria. We found 8 trials from high-income countries and 13 trials from low- and middle-income countries. Furthermore, at baseline, the health status of participants in 14 trials was healthy. Of the trials included, 7 of 21 used personality content, 12 of 21 used a 2-way communication strategy, and 7 of 21 used medical content. In the pooled analysis of 3937 participants (mean age: 35 years; 47.16%, 1857/3937 females), eHealth interventions significantly improved the ART adherence of people living with HIV (pooled Cohen *d*=0.25; 95% CI 0.05 to 0.46; *P*=.01). The interventions were also correlated with improved biochemical outcomes reported by 11 trials (pooled Cohen *d*=0.25; 95% CI 0.11 to 0.38; *P*<.001). The effect was sensitive to sample size (*Q*=5.56; *P*=.02) and study duration (*Q*=8.89; *P*=.003), but it could not be explained by other moderators. The primary meta-analysis result was stable in the 3 sensitivity analyses.

**Conclusions:**

Some of the eHealth interventions may be used as an effective method to increase the ART adherence of people living with HIV. Considering that most of the trials included a small sample size and were conducted for a short duration, these results should be interpreted with caution. Future studies need to determine the features of eHealth interventions to better improve ART adherence along with long-term effectiveness of interventions, effectiveness of real-time adherence monitoring, enhancement of study design, and influences on biochemical outcomes.

## Introduction

### Background

Owing to the significant role of antiretroviral therapy (ART) in controlling HIV from 2000 to 2017, AIDS-related deaths decreased by 38%, and approximately 11.4 million lives were saved [1]. Although ART has achieved great success, the 2030 treatment targets of the new 90-90-90 of the Joint United Nations Programme on HIV/AIDS appear unachievable for many countries [2]. In 2017, 21.7 million people living with HIV (PLWH) received ART, which accounted for only 59% of the global PLWH and 52% of children living with HIV [3]. In addition, according to the data from World Health Organization (WHO) in 2016, less than 50% of the PLWH achieved viral suppression [4]. Patients who strictly adhere to ART could control disease progression and prevent the emergence of drug-resistant mutations [5]. Poor ART adherence lead to the accelerated progression of PLWH to AIDS [6,7], increased demand for medical interventions [8], increased morbidity and mortality [9], and increased circulating ART-resistant strains [7,10]. A number of traditional measures can be used to ensure ART adherence, including behavioral skills training or medication adherence training, cognitive behavioral therapy, peer or social support, and counseling [11]. However, most of the interventions that are used in long-term therapy are either complicated or not widely applicable, and thus, more convenient, low-cost, and widely feasible innovations are required [8,12].

Owing to advances in mobile phone and internet technologies, the use of electronic health (eHealth) is expanding. The WHO Global Observatory for eHealth defines eHealth as “the use of information and communication technologies (ICT) for health” [13]. This involves the delivery of health information for health professionals and consumers through telecommunication (short message service, SMS; patient monitoring devices; and mobile phones) and internet-based components (social media, computer software, websites, mobile apps, games, and chat rooms) [14]. Numerous barriers to PLWH remain, including persistent stigma and discrimination [15], low socioeconomic status [16], punitive laws [17], and geographical isolation [18]. eHealth is increasingly used for the self-management and service delivery of HIV-related diseases [19]. eHealth interventions have many advantages: eHealth interventions are low cost and suitable for use in low- and middle-income countries (LMICs) [19-21] as well as convenient and accessible. According to estimates by the Ericsson 2018 Mobility Report, the number of mobile subscriptions worldwide will reach 7.9 billion in the third quarter of 2018 [22]. Moreover, popular social media platforms including WeChat [23], Facebook, and YouTube [24,25] have more than 1 billion monthly active users. In addition, eHealth can provide users with a private space to remove the discrimination and stigma associated with HIV [26,27]. eHealth can also boost behavioral changes, self-efficacy, knowledge, and clinical outcomes and has been developed for a wide range of disease and health behaviors [28-30].

In view of these advantages, an increasing number of reviews have studied the effects of eHealth on the promotion of ART adherence of PLWH. Therefore, in this study, before we conducted formal systematic literature search, a literature search was performed in MEDLINE to identify systematic reviews and meta-analyses published before March 20, 2018, that reviewed eHealth interventions to improve ART adherence (search terms are shown in Multimedia Appendix 1). Although favorable effects of eHealth interventions were documented, only narrative and systematic reviews were reported [31-34]. Moreover, additional reviews were either targeted to only 1 type of eHealth (such as SMS [7,35-37], social media [26], and voice calls [37]) or were only performed in the specific group of participants (men who have sex with men [38] and key populations in the Asia-Pacific region [19]).

### Objectives

With the publication of more and more studies focusing on ART adherence, this makes it possible to make quantitative and systematic assessments of the effectiveness and feasibility of eHealth interventions. In addition, despite the diversity of the interventions, we aggregated and compared their effects on improving ART adherence, which was supported by functional similarity and characteristics. So, the primary purpose of this study was to explore the effectiveness of eHealth interventions on improving ART adherence of PLWH. Moreover, the effects of different intervention characteristics, participant characteristics, and study characteristics were also assessed. To enhance the methodological quality of the meta-analysis and strengthen the conclusions, only randomized controlled trials (RCTs) were included.

## Methods

### Guidelines

This review was conducted following the Preferred Reporting Items for Systematic Reviews and Meta-Analyses statement [39] and the Cochrane Collaboration reporting items for systematic reviews and meta-analyses [40].

### Literature Search

We systematically searched MEDLINE (via PubMed), EMBASE, and the Cochrane Central Register of Controlled Trials for relevant studies published in English without restriction on publication date. The date of the last search for the electronic database was March 25, 2018. At the same time, we also searched for abstracts on several conference databases including the International AIDS Conference, the International AIDS Society Conference on HIV Science, and the Conference on Retroviruses and Opportunistic Infections. The reference lists of all relevant studies were searched manually to identify potential trials. The search strategy was developed by a librarian (LC) to identify studies that used eHealth interventions to improve ART adherence of PLWH. The study was developed based on Medical Subject Headings and key terms related to 4 categories: HIV, ART, adherence, and eHealth interventions. Detailed search items are listed in Multimedia Appendix 2.

### Selection Criteria

By following the populations, interventions, comparisons, outcomes, and study design (PICOS) framework, we included trials when (1) the study population was targeted to a sample of PLWH on ART; (2) the intervention focused only on eHealth interventions aimed to increase ART adherence rather than a data collection or participant recruitment tool; (3) the control group was the usual standard of care for PLWH; (4) the outcomes reported at least one ART adherence measurement (self-report, pill counting, electronic drug monitoring devices, or pharmacy refill record) and biochemical outcomes (viral load, log_10_ copies/mL, cluster of differentiation 4^+^ cell (CD4^+^) counting, or viral suppression [VS]/ virological failure [VF]); and (5) the study design was an RCT with a minimum of 3 months follow-up. No restrictions on the treatment of the participants, previous ART failure, or geography were applied. If multiple studies were reported on the same trial, the study with the most relevant outcome was included. Detailed PICOS criteria for the included studies are listed in Multimedia Appendix 3.

### Data Extraction

A total of 2 authors (ZW and YZ) independently reviewed all the titles and abstracts of the initial literature using bibliographic citation management software (EndNote, Version X7, Thomson Reuters) to determine their relevance based on the above-mentioned selection criteria. Relevant studies were kept for full-text reviews. Discrepancies were resolved by discussion with a third independent researcher (BQ).

Using a standardized extraction form (Microsoft Office Excel, Version 2013), the same 2 authors independently performed data extraction based on the following information: study characteristics (first author or research team, research year, setting, location, and study duration); participant characteristics (sample size [intervention arm, IA/control arm, CA], mean age, female ratio, and participant inclusion criteria); intervention characteristics (intervention type [IA/CA], frequency of intervention, intervention content [general content/medical content], personalization, and intervention communication strategy [1-way/2-way]); and outcome measures (primary adherence outcome measure [the proportion of medication taken as prescribed/the proportion of adherent patients], adherence outcome assessment methods, and biochemical outcome assessment methods). For studies with multiple IAs, eligible comparison trials were extracted and divided into distinct trials based on recent guidelines [40]. When a multiple-phase follow-up was reported, the outcome of the final follow-up corresponding to the study duration was used to assess the persistence and sustainability of the intervention [6,7]. The data of outcome measures were used to calculate the effect size in the meta-analysis. If there was insufficient data to calculate the effect size, the corresponding author was contacted by email. If the data were unavailable, studies were excluded [41]. For studies reporting a median and interquartile range for adherence outcomes, we converted the outcomes into the mean (SD) as previously reported [42,43].

### Assessment of Study Quality

Methodological quality is an important facet of this review. ZW and YZ independently assessed the risk of bias within individual included studies using the Cochrane risk-of-bias tool [40], which recommends 7 dimensions of research methodology for RCTs: (1) random sequence generation, (2) allocation concealment, (3) blinding of participants and personnel, (4) blinding of the outcome assessment, (5) incomplete outcome data, (6) selective outcome reporting, and (7) other sources of bias. The risk of bias for each item was evaluated at 3 levels: (1) high, (2) unclear, or (3) low. If a study was evaluated as a high or unclear risk of bias for sequence generation or randomization concealment, and other dimensions had more than 2 high risks of bias, the studies were considered as low overall quality. A third author (BQ) collated the results. Detailed quality assessments for the included studies are listed in Multimedia Appendix 4.

### Statistical Analysis

#### Statistical Methods

Statistical analyses of this meta-analysis were performed using the CMA Software (Comprehensive Meta-analysis, Version 2, Biostat). We used the mean effect size approach to pool estimates, which have been applied in other studies [7,8]. The effect size was weighted as per the study sample size. We calculated the odds ratio (OR) and the 95% CI for each included trial. Random-effects models were used to pool estimates as large between-study heterogeneity was expected. Cohen *d* values and the 95% CI were used to calculate the magnitude of the effect size. Values of 0.2, 0.5, and 0.8 were considered small, medium, and large effect sizes, respectively [8]. Reported *P* values were 2-tailed. To assess heterogeneity, I^2^ and Q statistics were used. I^2^ statistic exceeding 50% with a significant *Q* value (*P*<.05) represented substantial heterogeneity [44]. I^2^ also represented the levels of heterogeneity with values of 25%, 50%, and 75% indicating low, moderate, and high heterogeneity, respectively [45]. Funnel plot symmetry [46] and Egger regression intercept [47] were used to assess publication bias. If publication bias existed, the funnel plots were asymmetric (Egger test: *P*<.05). We used trim-and-fill analysis described by Duval and Tweedie to estimate the number of missing studies because of publication bias and calculated the effect size after correction [48].

Weighted mean effect sizes were calculated to estimate the overall difference between eHealth and control groups on adherence outcomes as well as on biochemical outcomes because biochemical outcomes are the final presentation of adherence. Of the trials that reported different outcomes, the majority of trials (14/21) reported multiple adherence outcomes, and more than half of the trials (6/11) reported multiple biochemical outcomes. Considering that multiple effect sizes in 1 trial violated the independence assumption in meta-analysis, we selected only 1 effect size for each trial in our analyses. When trials had multiple outcome assessment methods, we selected the most objective and reliable method according to a predetermined order (assessed in the following order: electronic monitoring, pill counting, pharmacy refill record, self-report, and treatment interruption; viral load, log_10_ copies/mL, CD4^+^ cell counting, and VS/VF; and continuous scale over dichotomized scale) as used in other studies [8,49]. The mean effect size was also independently calculated in all the adherence assessment methods and the biochemical outcome assessment methods of the trials.

#### Subgroup Analyses

Given the potential for substantial significant heterogeneity across the studies (based on the I^2^ and Q statistics for heterogeneity), we performed subgroup analyses to explore the potential factors that moderate the overall effect size. The following moderators were examined: age (age <36.65 years or age ≥36.65 years), study duration (short-term trial: duration ≤36 weeks or long-term trial: duration >36 weeks), sample size (large trial: n≥166 or small trial: n<166), location (high-income countries or LMICs), participant ART status at baseline (nonadherence, ART-naïve, or treatment experienced), participant health status at baseline (healthy or at risk), age category (adults or adults and adolescents), primary outcome measure (proportion of medication taken as prescribed or proportion of patients with good adherence), type of intervention (Web-based computer programs, telephone calls, SMS, electronic adherence monitoring device [EAMD], or SMS plus telephone calls), frequency of intervention (real-time, daily, or frequency below daily), intervention content (medical content or general content), communication strategy (1-way or 2-way), and personalization (yes or no). In particular, we also divided the type of intervention into telecommunication subgroup and internet-based component subgroup so that we could explore whether there was a notable and significant difference between the 2 subgroups. The cut-off points for moderators (age, sample size, and study duration) were based on the median values among trials from the available information, which was used by several previous studies [8,49].

#### Sensitivity Analyses

A total of 3 sensitivity analyses were performed to assess stability of the meta-analysis. The first sensitivity analysis excluded low-quality trials, the second excluded trials with a sample attrition rate ≥20%, and the third gave higher weight to specific assessment methods (self-report and CD4^+^ cell counting) for the trials reporting multiple outcome measures.

## Results

### Study Characteristics

**Figure 1:**
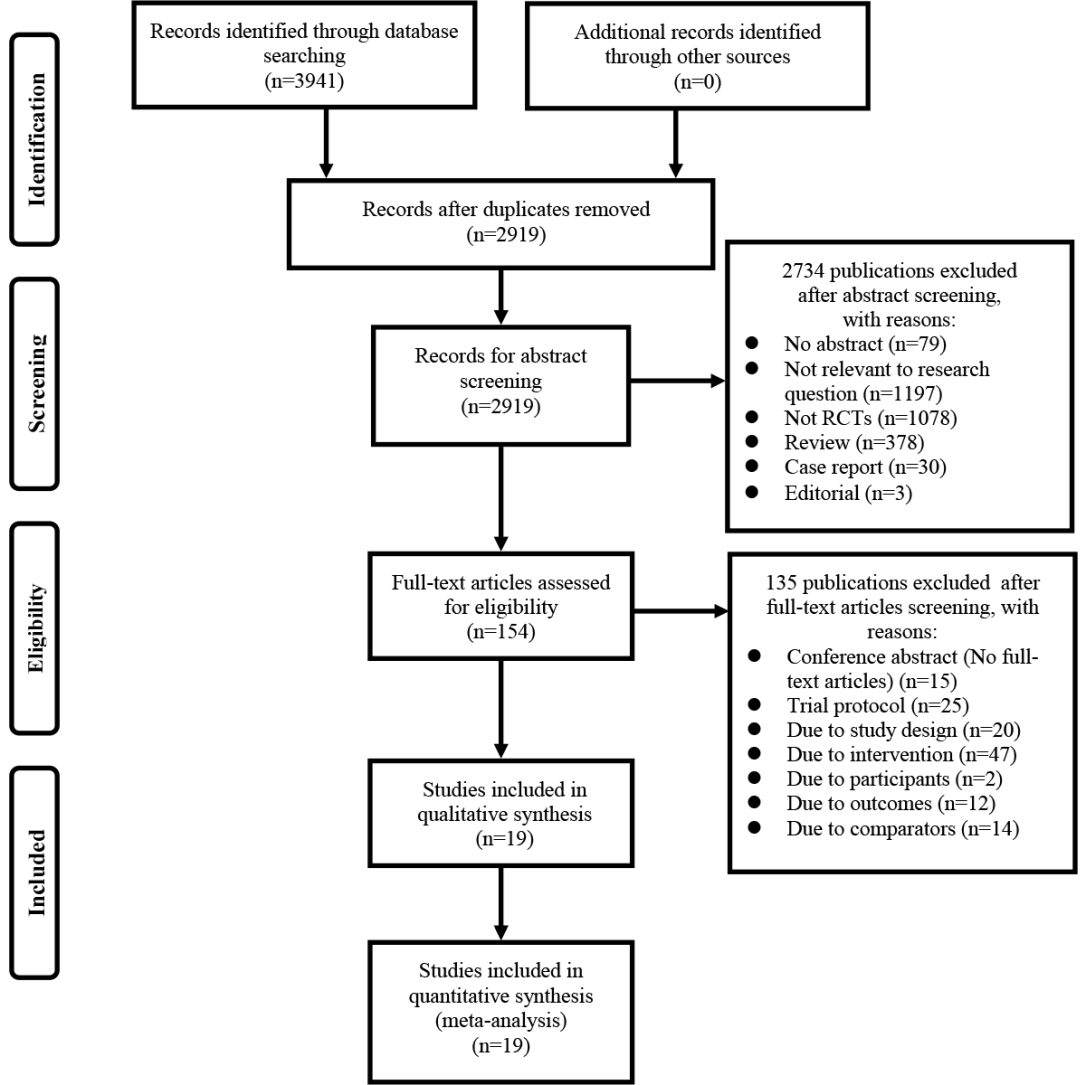
Preferred Reporting Items for Systematic Reviews and Meta-Analyses chart showing study selection process. RCT: randomized controlled trial.

A total of 19 RCTs were identified following the assessment of 154 full-text articles (Figure 1; Multimedia Appendices 5-8) [50-68]. We extracted 2 independent comparison trials (daily SMS and weekly SMS) from the study of Pop-Eleches et al [54] and 2 comparison trials (1-way and 2-way communication strategies) from the study of Linnemayr et al [68]. Finally, a total of 21 trials were included in the meta-analysis consequently. A total of 21 trials included 3937 participants. The sample size varied from 21 to 631, with a median of 166. The mean age of the participants was 35 years (Safren et al failed to report the mean age of participants [50]), and 47.16% (1857/3937) were female. Studies were performed in the United States [50-52,57,59,61,62,64], Kenya [53,54], China [60,65], Uganda [68], Brazil [56], India [58], Cameroon [55], South Africa [63], Botswana [66], and Malaysia [67]. Study duration ranged from 12 to 96 weeks, with a median of 36 weeks. One-third of the trials targeted at-risk populations (7/21), 43% focused on ART-naïve populations (9/21), 76% focused on adults (16/21), and the remainder focused on adults and adolescents (5/21).

The purpose of the included studies was to improve ART adherence of PLWH. Self-report (10/21) and electronic drug monitoring device (medication event monitoring system cap and EAMD [Wisepill]; 10/21) were the most commonly used methods to assess adherence, followed by pill counting (2/21), pharmacy refill record (2/21), or treatment interruption (4/21). Primary type of outcome measure was presented as the proportion of medication taken as prescribed in 15 trails and as the proportion of patients with good adherence in 6 trials. Biochemical outcomes were measured through CD4^+^ cell counting (6/21), viral load, log_10_ copies/mL (5/21), and VS/VF (6/21).

### Electronic Health Characteristics

The eHealth characteristics are varied across the 21 trials. A total of 12 trials sent SMSs, 4 used telephone calls, 2 performed interventions in Web-based computer program, 2 used EAMD, and 1 combined SMS with telephone calls. According to another classification method of intervention, 19 trials were divided into telecommunication subgroup, and the remaining 2 trials were divided into internet-based component subgroup. A total of 19 trials conducted interventions at a fixed predetermined frequency (daily or frequency below daily), and the remaining 2 trials used real-time medication monitoring in which the participants were sent a reminder if they did not open the medication management device within the specified time. The intervention content was general content in 14 trials (medication reminders, humor jokes, as well as motivation and encouragement) and medical content in 7 trials (HIV/AIDS-related knowledge, the importance of adherence, and skills for good adherence). Moreover, 12 trials supported a 2-way communication strategy (patients were permitted, encouraged, or demanded to respond to the received information). Furthermore, 7 trials used personalized content (eg, the trial by Simoni et al [52] used the flexible content of messages to accommodate the different needs and schedules of the participants).

### Meta-Analyses

Table 1 shows the mean effect sizes across all types of outcome assessment methods. Statistical significance for the individual outcome assessment method was not always achieved because of the limited statistical power of the available studies. For the 5 adherence outcome assessment methods, significant results in both self-report (*k=*10; Cohen *d=*0.44; 95% CI 0.11 to 0.77; *P*=.01) and pharmacy refill record (*k=*2; Cohen *d=*0.47; 95% CI 0.11 to 0.84; *P*=.01) were observed. For the 3 biochemical outcome assessment methods, CD4^+^ cell counting had a small positive effect size (*k=*6; Cohen *d=*0.20; 95% CI 0.04 to 0.35; *P*=.01), and viral load (log_10_ copies/mL) had a negative significant effect size (*k=*5; Cohen *d=*−0.40; 95% CI −0.62 to −0.17; *P*<.001).

**Table 1:** The effect of electronic health on antiretroviral therapy adherence outcomes and biochemical outcomes by type of outcome assessing methods.

Measures	*k* (number of trials)	Odds ratio (95% CI)	Cohen *d* (95% CI)	*P* value	I^2^ (%)
Electronic drug monitoring device	10	1.20 (0.75 to 1.93)	0.10 (−0.16 to 0.36)	.46	80.44
Self-report	10	2.20 (1.21 to 4.00)	0.44 (0.11 to 0.77)	.01	88.52
Pill counting	2	0.79 (0.52 to 1.21)	−0.13 (−0.36 to 0.10)	.28	2.82
Pharmacy refill record	2	2.36 (1.22 to 4.56)	0.47 (0.11 to 0.84)	.01	0.00
Treatment interruption	4	0.69 (0.41 to 1.15)	−0.21 (−0.49 to 0.08)	.15	0.00
Cluster of differentiation 4^+^ cell counting	6	1.43 (1.08 to 1.89)	0.20 (0.04 to 0.35)	.01	21.94
Viral load (log_10_ copies/mL)	5	0.49 (0.32 to 0.73)	−0.40 (−0.62 to −0.17)	<.001	30.83
Viral suppression/virological failure	6	1.32 (0.90 to 1.93)	0.15 (−0.06 to 0.36)	.16	34.53
Mean biochemical outcomes	11	1.57 (1.22 to 2.01)	0.25 (0.11 to 0.38)	<.001	43.16
Mean adherence outcomes	21	1.59 (1.10 to 2.29)	0.25 (0.05 to 0.46)	.01	86.70

**Figure 2:**
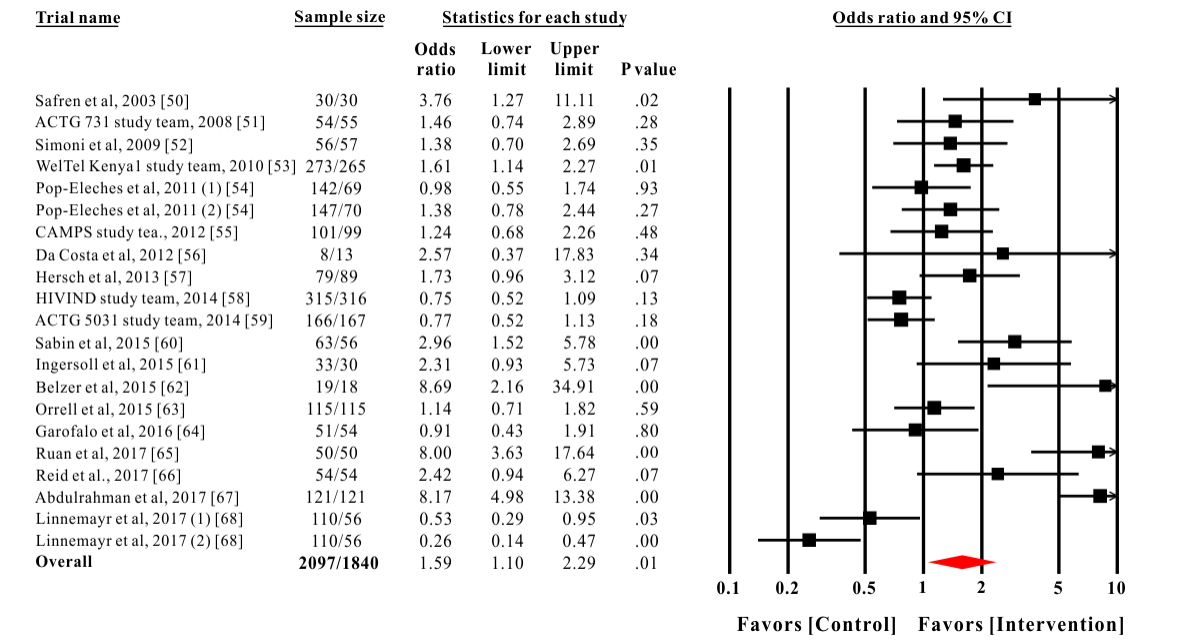
The effect of electronic health intervention on antiretroviral therapy adherence of people living with HIV. Two independent comparison trials (daily short message service [SMS] and weekly SMS) from the study of Pop-Eleches et al were extracted as Pop-Eleches et al (1) and Pop-Eleches et al (2), and two independent comparison trials (1-way and 2-way communication strategies) from the study of Linnemayr et al were extracted as Linnemayr et al (1) and Linnemayr et al (2).

**Figure 3:**
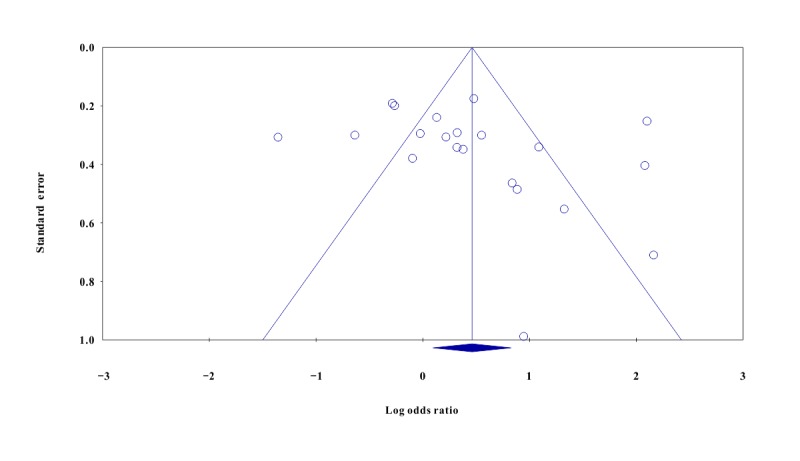
Funnel plot of SE and log odds ratio on antiretroviral therapy adherence of people living with HIV between intervention and control groups.

In the pooled analysis of 21 trials, eHealth interventions significantly improved ART adherence (OR=1.59, 95% CI 1.10 to 2.29; *P*=.01; Figure 2). The weighted mean effect size (Cohen *d*) was 0.25 (95% CI 0.05 to 0.46). A small positive effect of eHealth interventions on improving ART adherence of PLWH was observed. Heterogeneity assessments showed variability across the trials (*Q*_20_=150.36; *P*<.001). There was high heterogeneity (I^2^: 86.70%) across trials, which supported the selection of the random-effects model to perform subgroup analyses to investigate the impact of the moderators on the overall effect size. Publication bias was not detected through funnel plot analysis (Figure 3) and Egger regression tests (Intercept of the regression line: 2.39; 95% CI –1.12 to 5.91; t_19_=1.43; *P*=.17). Duval and Tweedie’s trim-and-fill analysis showed that no studies were trimmed or filled, indicating no evidence of publication bias. In addition, biochemical outcomes reported by 11 trials also had a weighted mean effect size that achieved statistical significance (Cohen *d*=0.25; 95% CI 0.11 to 0.38; *P*<.001; Table 1).

### Subgroup Analyses

The study and participant characteristics could explain some heterogeneity across the trials, specifically the sample size and study duration (Table 2). The subgroup analysis of sample size showed that large trials (Cohen *d*=0.06; 95% CI −0.20 to 0.33) had smaller effect sizes than small trials (Cohen *d*=0.51; 95% CI 0.25 to 0.76), which showed a significant difference in ART adherence between these 2 subgroups (*Q*=5.56; *P*=.02). Short-term trials displayed medium effect sizes (Cohen *d*=0.51; 95% CI 0.23 to 0.79), whereas long-term trials showed no significant effect size (Cohen *d*=−0.01; 95% CI −0.22 to 0.19), which indicated a significant difference in ART adherence between the 2 subgroups (*Q*=8.89; *P*=.003). However, heterogeneity cannot be explained by mean age, location, participant ART status at baseline, participant health status at baseline, age category, primary type of outcome measure, and all the eHealth interventions characteristics (Tables 2 and 3).

**Table 2:** Subgroup analyses of the effect of electronic health on antiretroviral therapy adherence by study and participant characteristics.

Moderator and subgroups		*k* (number of trials)	Odds ratio (95% CI)	*Q* value	*P* value for heterogeneity
**Sample size**				5.59	.02
	Large trial	11	1.12 (0.70 to 1.81)		
	Small trial	10	2.50 (1.58 to 3.97)		
**Mean age (years)**				0.58	.45^a^
	<36.65	9	1.28 (0.61 to 2.70)		
	≥36.65	11	1.76 (1.25 to 2.49)		
	Not specified	1	3.76 (1.23 to 11.48)		
**Study duration**				8.89	.003
	Short-term trial	11	2.52 (1.53 to 4.16)		
	Long-term trial	10	0.98 (0.67 to 1.42)		
**Location**				0.03	.86
	High-income countries	8	1.62 (1.04 to 2.53)		
	Low- and middle-income countries	13	1.52 (0.91 to 2.55)		
**Participant ART^b^ status at baseline**				4.48	.11
	ART-naïve	9	1.68 (0.98 to 2.89)		
	Nonadherence	6	2.55 (1.39 to 4.66)		
	Treatment experienced	6	0.97 (0.50 to 1.88)		
**Participant health status at baseline**				2.07	.15
	At risk	7	2.24 (1.34 to 3.73)		
	Healthy	14	1.36 (0.87 to 2.12)		
**Age category**				2.73	.10
	Adults	16	1.89 (1.29 to 2.78)		
	Adults and adolescents	5	0.89 (0.40 to 1.99)		
**Primary type of outcome measure**				0.92	.34
	Proportion of medication taken as prescribed	15	1.71 (1.03 to 2.86)		
	Proportion of patients with good adherence	6	1.26 (0.87 to 1.83)		

^a^The trial by Safren et al [50] did not report the mean age of participants.

^b^ART: antiretroviral therapy.

**Table 3:** Subgroup analyses of the effect of electronic health on antiretroviral therapy adherence by intervention characteristics.

Moderator and subgroup		*k* (number of trials)	Odds ratio (95% CI)	*Q* value	*P* value for heterogeneity
**Type of intervention^a^**				2.12	.55^b^
	Web-based computer program	2	2.22 (1.09 to 4.51)		
	Telephone call	4	1.21 (0.66 to 2.22)		
	SMS^c^	12	1.31 (0.83 to 2.06)		
	Electronic adherence monitoring device	2	1.78 (0.70 to 4.54)		
	SMS plus telephone call	1	8.17 (4.98 to 13.38)		
**Type of intervention^d^**				0.83	.36
	Telecommunication	19	1.53 (1.03 to 2.25)		
	Internet-based component	2	2.22 (1.09 to 4.51)		
**Frequency of intervention**				0.29	.86
	Real-time	2	1.78 (0.70 to 4.54)		
	Daily	7	1.72 (1.10 to 2.70)		
	Frequency below daily	12	1.44 (0.83 to 2.50)		
**Intervention content**				0.19	.67
	General content	14	1.50 (0.93 to 2.42)		
	Medical content	7	1.77 (0.97 to 3.23)		
**Communication strategy**				0.67	.41
	1-way	9	1.91 (1.00 to 3.64)		
	2-way	12	1.38 (0.90 to 2.13)		
**Personalization**				0.89	.34
	Yes	7	1.31 (0.93 to 1.84)		
	No	14	1.78 (1.04 to 3.07)		

^a^The intervention was divided into 5 subgroups: Web-based computer program, telephone call, short message service (SMS), electronic adherence monitoring device, and SMS plus telephone call.

^b^The trial by Abdulrahman et al [67] was the only one that used SMS plus telephone calls.

^c^SMS: short message service.

^d^The intervention was divided into 2 subgroups: telecommunication and internet-based component.

### Sensitivity Analyses

The primary meta-analysis result was stable in the 3 sensitivity analyses. The effect of eHealth on improving ART adherence of PLWH did not change when we excluded 4 trials [50,51,61,63] with low quality (Cohen *d*=0.24; 95% CI 0.00 to 0.48; *P*=.049), when we excluded a trial [50] with sample attrition rates ≥20% (Cohen *d*=0.24; 95% CI 0.03 to 0.44; *P*=.02), and when we gave higher weight to self-report (replace the adherence assessment method of 2 trials—Simoni et al [52] and da Costa et al [56]—with the self-report; Cohen *d*=0.26; 95% CI 0.05 to 0.46; *P*=.01). In addition, the effect of eHealth on the biochemical outcomes of PLWH did not change when we gave higher weight to CD4^+^ cell counting (replace the viral load, log_10_ copies/mL, of 3 trials—Simoni et al [52], Reid et al [66], and Abdulrahman et al [67]—with the CD4^+^ cell counting; Cohen *d*=0.20; 95% CI 0.08 to 0.32; *P*<.001).

## Discussion

### Principal Findings

This review identified 21 trials of 19 RCTs that investigated the effectiveness of eHealth interventions on improving ART adherence of PLWH. Overall, eHealth interventions reported significant, but small, positive effects on ART adherence (Cohen *d*=0.25; 95% CI 0.05 to 0.46; *P*=.01) compared with PLWH in usual care. This finding was also stable in 3 sensitivity analyses. Specifically, SMS, telephone call, and EAMD were not able to significantly increase ART adherence of PLWH; however, the study of combining SMS and telephone call was highly effective in improving ART adherence. In addition, the Web-based computer program also showed significant positive effects in ART adherence. Our review also found that both telecommunication and internet-based components reported significant positive effects on ART adherence of PLWH. This meta-analysis result demonstrated that some of eHealth interventions showed favorable effects to improve ART adherence of PLWH, which was consistent with findings of previous reviews. Daher et al [14] found that the digital innovations (mobile health, mHealth; internet-based mHealth/eHealth; and combined innovations) reported strong positive effects on improving ART adherence and clinic attendance rates. However, although significant, the small effect detected in this review was not sufficient to improve ART adherence and make it to the satisfactory clinical standard. Conn et al [69] mentioned in their study that the patient’s medication adherence is difficult to change. The reasons for the ART nonadherence of PLWH are very complicated. In addition to the reason for forgetting to take medicine [70], it may also include psychological factors found in early reviews, such as depressive symptoms [70], stigma [71], and lack of social support [15]. Previous studies suggested that patients with chronic diseases may develop negative emotions during long-term medication and believe that their illnesses are incurable so that lacks the motivation to adhere to medication [12]. Moreover, nonadherence is also related to many factors including medication burden [72], side effects [73], and socioeconomic status [74]. Future research could try to use eHealth, educational, and psychosocial interventions together to better improve the ART adherence of PLWH.

### Moderators on the Use of Electronic Health Interventions

Although this meta-analysis did not have significant publication bias, we noted significant heterogeneity, which may be because of clinical heterogeneity (the real difference of the impact generated from the different eHealth interventions and participant populations) or methodological heterogeneity (the difference generated from the different outcome assessment methods defined and measured in each study) [8]. Previous reviews on ART adherence also reported high heterogeneity [41]. Subgroup analyses showed that the effectiveness of eHealth interventions was sensitive to sample size and study duration. We found that small trials with limited sample sizes reported larger beneficial effects than large trials, which could be explained by the *small-study effects* proposed by Sterne et al [75]. Previous studies found that intervention effects were exaggerated in small trials with inadequate or unclear sequence generation, inadequate or unclear allocation concealment, and lack of blinding [76,77]. This is consistent with our findings that most of the trials with unclear sequence generation were small trials (4/5), and most of the small trials had unclear or high risk with blinding (9/10). The results of these small trials might overestimate the true effect of the interventions, and this effect is more easily published. Therefore, we should explain the results of the small trials with caution. Our subgroup analyses also indicated a higher effect size for short-term trials compared with long-term trials. This suggested that the effects of the eHealth interventions weakened over time. This finding is consistent with the findings of the study by Vervloet et al [78] who suggested that electronic reminders led to short-term improvements of the patients’ adherence to medication, but the long-term effects were unclear. This finding has important clinical significance because the long-term effectiveness of eHealth interventions is a recent focus of attention. In addition, for the trials included in this review, most of them (19/21) were eHealth interventions with a fixed frequency. These trials automatically sent eHealth reminders regardless of whether or not patients took the medications. As patients become familiar with reminders, they will gradually become habitualized and generate response fatigue to the eHealth intervention, which may have a negative impact on the long-term effectiveness of interventions. Some of the trials in this review focus on real-time adherence monitoring, which only provides intervention when the patients fail to take the medicine on time, thus avoiding habitualization of reminders [60,63]. Although the 2 trials did not find a significant pooled effect of real-time reminders, it should be noted that the number of available studies limited statistical power. Future adherence intervention studies should strengthen study design in both sequence generation and blinding and should focus on real-time adherence monitoring to enhance the long-term effectiveness of eHealth interventions.

Another interesting area of this review is the effects of the eHealth interventions characteristics. As the number of available studies in some subgroups limited statistical power, the results should be considered uncertain, so we recommend that the comparison between these subgroups should be interpreted with caution. SMS did not have a significant effect on improving ART adherence in this review; however, the result was inconsistent with the result of the study by Finitsis et al [7]. This may be because SMSs are facing challenges from internet protocol–based messaging services in recent years (such as Apple’s iMessage, WhatsApp, Facebook Messenger, WeChat, and Line). Therefore, the attention and use of high-cost SMSs are gradually decreasing. People’s reactions to application-to-peer messaging in their daily lives have also weakened. Although previous studies have suggested that the outcomes can be improved by changing certain intervention characteristics (eg, increasing the frequency of the intervention [78] and performing 2-way communications [79]), no significant heterogeneity between these subgroups was observed in this review. Further research could use the “nudge theory” to guide the design of the eHealth interventions procedure for improving ART adherence of PLWH. The theory emphasizes that nudges are not mandatory, and their intervention design must be simple and inexpensive [80]. It was explored in previous studies that this theory had a positive impact on several behaviors, such as reducing tobacco use [81], changing adult dietary choices [82], and increasing physical activity [83]. For medication reminders, any intervention that directly asks participants about trial content should be excluded, as this would bias the participants.

### Selection of Adherence Outcome Assessment Method

Although an array of methods are proposed to assess adherence, few meet the gold standards of reliability, ease of use, low cost, flexibility, and practicality. However, each method has its advantages and disadvantages. According to Lam and Fresco, subjective methods can generally explain nonadherence, whereas objective methods can more accurately measure patient adherence to medication [84]. Subjective methods have the advantage of low cost, simplicity, practicality, and flexibility. However, poor sensitivity and specificity remain an issue, and questionnaires are unreliable in terms of adherence outcomes. The patient’s psychological state can also influence the accuracy of the outcomes. Outcomes are more accurate for objective methods than those for subjective methods. However, different objective methods have variable characteristics. Although pill counting is simple and low cost, it fails to identify the medication-taking pattern. Electronic monitoring devices are only suitable for small-scale research as expensive technical support is required. Considering both accuracy and cost, pharmacy refill record is more beneficial for large numbers of research populations [85]. The assessment of biochemical outcomes can directly reflect overall treatment regimens and indirectly reflect the effectiveness of the interventions. However, these methods are expensive and intrusive. Considering the advantages and disadvantages of various outcome assessment methods, we recommend that these methods should be applied in combination in future research according to the characteristics of each study to achieve measurement purpose.

### Strengths and Limitations

This review has several strengths. First, this review only includes RCTs, which are considered to enhance the methodological quality of the meta-analysis and strengthen the conclusions. Another strength is that the results of our meta-analysis indicated no influence of publication bias. In addition, subgroup analyses were performed to explore the source of between-study heterogeneity. We examined numerous moderators that significantly contribute to the design and implementation of eHealth interventions. Moreover, we performed some sensitivity analyses to detect the robustness of our results.

We found that the included studies on eHealth interventions had several limitations. First, of the 19 studies, 15 had a high or unclear risk of bias for at least one of the bias items in the methodological quality assessment. The low quality of the studies may bias the meta-analysis and reduce further pooled analysis [26]. Moreover, some of the primary outcome measures were expressed by the proportion of patients with good adherence. The level of adherence that was defined as “good” differed across the trails (thresholds were 90% in 2 trials, 95% in 4 trials, and 100% in 1 trial). Low thresholds may overestimate the effectiveness of eHealth on ART adherence [8].

Several limitations of this review should also be considered when we interpret the findings. The findings are inevitably limited by the number of studies in some moderators in the subgroup analyses that make it difficult to generalize their results. Several moderators examined in subgroup analyses may also impact each other, so they should be interpreted with caution. In addition, although we calculated Cohen *d* to standardize these measures, methodological disadvantages were observed when the adherence measures were pooled [49]. Furthermore, although the design of the RCT can provide strong evidence, it is precisely because of the rigorous randomization, blinding, quality control, and other design in the RCT that the effect of the research often deviates from the actual effect in the “real world.” Finally, we restricted the study of English language publications, and further studies across a range of ethnicities would further strengthen the findings.

### Conclusions

We found that some of the eHealth interventions may be the effective method to increase the ART adherence of PLWH. The advantages of low cost, ease of access, and confidentiality make it a useful intervention tool in the PLWH. Although our analyses suggest some heterogeneity across trials, this finding is likely because of variation in the characteristics of the studies and in the definitions of outcomes among the studies. Considering that most of the trials are with small sample sizes or short-term duration, these results should be interpreted with caution. Therefore, the effectiveness of eHealth interventions in the “real world” remains uncertain.

To better identify the role of eHealth interventions in improving ART adherence of PLWH, future research needs to determine the features of eHealth interventions to better improve ART adherence along with long-term effectiveness of interventions, effectiveness of real-time adherence monitoring, enhancement of study design, and influences on biochemical outcomes. In addition, further research can try to design and implement the optimal strategy of eHealth intervention based on *nudge theory* combined with educational and psychosocial interventions.

## Appendix

Multimedia Appendix 1 Reviews literature search strategy.

Multimedia Appendix 2 Formal systematic literature search strategy.

Multimedia Appendix 3 Population, interventions, comparisons, outcomes and study design (PICOS) criteria for study inclusion.

Multimedia Appendix 4 Cochrane risk of bias quality assessment for included studies.

Multimedia Appendix 5 List of included studies after full-text review.

Multimedia Appendix 6 List of excluded studies after full-text review.

Multimedia Appendix 7 Study and participants characteristics of trials for principal systematic literature review.

Multimedia Appendix 8 Characteristics of electronic health intervention and outcome measures of trials for principal systematic literature review.

## Abbreviations

ART: antiretroviral therapy
CA: control arm
CD4^+^: cluster of differentiation 4^+^
EAMD: electronic adherence monitoring device
eHealth: electronic health
IA: intervention arm
LMICs: low- and middle-income countries
mHealth: mobile health
OR: odds ratio
PICOS: populations, interventions, comparisons, outcomes, and study design
PLWH: people living with HIV
RCTs: randomized controlled trials
SMS: short message service
VF: virological failure
VS: viral suppression
WHO: World Health Organization

## References

[1] (2019). World Health Organization.

[2] (2017). The Joint United Nations Programme on HIV/AIDS (UNAIDS).

[3] (2018). World Health Organization.

[4] (2017). The Joint United Nations Programme on HIV/AIDS (UNAIDS).

[5] Hamine S, Gerth-Guyette E, Faulx D, Green BB, Ginsburg AS (2015). Impact of mhealth chronic disease management on treatment adherence and patient outcomes: a systematic review. J Med Internet Res.

[6] Sherr L, Lampe FC, Clucas C, Johnson M, Fisher M, Date HL, Anderson J, Edwards S, Smith CJ, Hill T, Harding R (2010). Self-reported non-adherence to ART and virological outcome in a multiclinic UK study. AIDS Care.

[7] Finitsis DJ, Pellowski JA, Johnson BT (2014). Text message intervention designs to promote adherence to antiretroviral therapy (ART): a meta-analysis of randomized controlled trials. PLoS One.

[8] Thakkar J, Kurup R, Laba TL, Santo K, Thiagalingam A, Rodgers A, Woodward M, Redfern J, Chow CK (2016). Mobile telephone text messaging for medication adherence in chronic disease: a meta-analysis. JAMA Intern Med.

[9] Ho PM, Magid DJ, Shetterly SM, Olson KL, Maddox TM, Peterson PN, Masoudi FA, Rumsfeld JS (2008). Medication nonadherence is associated with a broad range of adverse outcomes in patients with coronary artery disease. Am Heart J.

[10] Ferreira JL, Rodrigues R, Lança AM, de Almeida VC, Rocha SQ, Ragazzo TG, Estevam DL, Brigido LF (2013). Transmitted drug resistance among people living with HIV/Aids at major cities of Sao Paulo state, Brazil. Adv Virol.

[11] Kanters S, Park JJ, Chan K, Socias ME, Ford N, Forrest JI, Thorlund K, Nachega JB, Mills EJ (2017). Interventions to improve adherence to antiretroviral therapy: a systematic review and network meta-analysis. Lancet HIV.

[12] Nieuwlaat R, Wilczynski N, Navarro T, Hobson N, Jeffery R, Keepanasseril A, Agoritsas T, Mistry N, Iorio A, Jack S, Sivaramalingam B, Iserman E, Mustafa RA, Jedraszewski D, Cotoi C, Haynes RB (2014). Interventions for enhancing medication adherence. Cochrane Database Syst Rev.

[13] World Health Organization (2016). Global Diffusion of eHealth: Making Universal Health Coverage Achievable: Report of the Third Global Survey on eHealth.

[14] Daher J, Vijh R, Linthwaite B, Dave S, Kim J, Dheda K, Peter T, Pai NP (2017). Do digital innovations for HIV and sexually transmitted infections work? Results from a systematic review (1996-2017). BMJ Open.

[15] Katz IT, Ryu AE, Onuegbu AG, Psaros C, Weiser SD, Bangsberg DR, Tsai AC (2013). Impact of HIV-related stigma on treatment adherence: systematic review and meta-synthesis. J Int Aids Soc.

[16] Burch LS, Smith CJ, Phillips AN, Johnson MA, Lampe FC (2016). Socioeconomic status and response to antiretroviral therapy in high-income countries: a literature review. AIDS.

[17] Lehman JS, Carr MH, Nichol AJ, Ruisanchez A, Knight DW, Langford AE, Gray SC, Mermin JH (2014). Prevalence and public health implications of state laws that criminalize potential HIV exposure in the United States. AIDS Behav.

[18] Kagee A, Remien RH, Berkman A, Hoffman S, Campos L, Swartz L (2011). Structural barriers to ART adherence in Southern Africa: challenges and potential ways forward. Glob Public Health.

[19] Purnomo J, Coote K, Mao L, Fan L, Gold J, Ahmad R, Zhang L (2018). Using ehealth to engage and retain priority populations in the HIV treatment and care cascade in the Asia-Pacific region: a systematic review of literature. BMC Infect Dis.

[20] Tran BX, Houston S (2012). Mobile phone-based antiretroviral adherence support in Vietnam: feasibility, patient's preference, and willingness-to-pay. AIDS Behav.

[21] Rodrigues R, Bogg L, Shet A, Kumar DS, de Costa A (2014). Mobile phones to support adherence to antiretroviral therapy: what would it cost the Indian National AIDS Control Programme?. J Int AIDS Soc.

[22] (2018). Ericsson - A World of Communication.

[23] (2018). Statista.

[24] Alzougool B (2018). The impact of motives for Facebook use on Facebook addiction among ordinary users in Jordan. Int J Soc Psychiatry.

[25] (2018). Statista.

[26] Cao B, Gupta S, Wang J, Hightow-Weidman LB, Muessig KE, Tang W, Pan S, Pendse R, Tucker JD (2017). Social media interventions to promote HIV testing, linkage, adherence, and retention: systematic review and meta-analysis. J Med Internet Res.

[27] Rao D, Frey S, Ramaiya M (2018). eHealth for stigma reduction efforts designed to improve engagement in care for people living with HIV. Curr HIV/AIDS Rep.

[28] Taylor GM, Dalili MN, Semwal M, Civljak M, Sheikh A, Car J (2017). Internet-based interventions for smoking cessation. Cochrane Database Syst Rev.

[29] Alexander GL, McClure JB, Calvi JH, Divine GW, Stopponi MA, Rolnick SJ, Heimendinger J, Tolsma DD, Resnicow K, Campbell MK, Strecher VJ, Johnson CC, MENU Choices Team (2010). A randomized clinical trial evaluating online interventions to improve fruit and vegetable consumption. Am J Public Health.

[30] Schnall R, Travers J, Rojas M, Carballo-Diéguez A (2014). eHealth interventions for HIV prevention in high-risk men who have sex with men: a systematic review. J Med Internet Res.

[31] Lima IC, Galvão MT, Alexandre HD, Lima FE, Araújo TL (2016). Information and communication technologies for adherence to antiretroviral treatment in adults with HIV/AIDS. Int J Med Inform.

[32] Jongbloed K, Parmar S, van der Kop M, Spittal PM, Lester RT (2015). Recent evidence for emerging digital technologies to support global HIV engagement in care. Curr HIV/AIDS Rep.

[33] Campbell JI, Haberer JE (2015). Cell phone-based and adherence device technologies for HIV care and treatment in resource-limited settings: recent advances. Curr HIV/AIDS Rep.

[34] Simoni JM, Kutner BA, Horvath KJ (2015). Opportunities and challenges of digital technology for HIV treatment and prevention. Curr HIV/AIDS Rep.

[35] Horvath T, Azman H, Kennedy GE, Rutherford GW (2012). Mobile phone text messaging for promoting adherence to antiretroviral therapy in patients with HIV infection. Cochrane Database of Systematic Reviews.

[36] Mbuagbaw L, Mursleen S, Lytvyn L, Smieja M, Dolovich L, Thabane L (2015). Mobile phone text messaging interventions for HIV and other chronic diseases: an overview of systematic reviews and framework for evidence transfer. BMC Health Serv Res.

[37] Amankwaa I, Boateng D, Quansah DY, Akuoko CP, Evans C (2018). Effectiveness of short message services and voice call interventions for antiretroviral therapy adherence and other outcomes: a systematic review and meta-analysis. PLoS One.

[38] Muessig KE, LeGrand S, Horvath KJ, Bauermeister JA, Hightow-Weidman LB (2017). Recent mobile health interventions to support medication adherence among HIV-positive MSM. Curr Opin HIV AIDS.

[39] Hutton B, Salanti G, Caldwell DM, Chaimani A, Schmid CH, Cameron C, Ioannidis JP, Straus S, Thorlund K, Jansen JP, Mulrow C, Catalá-López F, Gøtzsche PC, Dickersin K, Boutron I, Altman DG, Moher D (2015). The PRISMA extension statement for reporting of systematic reviews incorporating network meta-analyses of health care interventions: checklist and explanations. Ann Intern Med.

[40] Higgins JP, Green S (2008). Cochrane Handbook for Systematic Reviews of Interventions.

[41] Spaan P, van Luenen S, Garnefski N, Kraaij V (2018). Psychosocial interventions enhance HIV medication adherence: A systematic review and meta-analysis. J Health Psychol.

[42] Luo D, Wan X, Liu J, Tong T (2018). Optimally estimating the sample mean from the sample size, median, mid-range, and/or mid-quartile range. Stat Methods Med Res.

[43] Wan X, Wang W, Liu J, Tong T (2014). Estimating the sample mean and standard deviation from the sample size, median, range and/or interquartile range. BMC Med Res Methodol.

[44] Higgins JP, Thompson SG (2002). Quantifying heterogeneity in a meta-analysis. Stat Med.

[45] Higgins JP, Thompson SG, Deeks JJ, Altman DG (2003). Measuring inconsistency in meta-analyses. Br Med J.

[46] Cooper H, Hedges LV (1997). The Handbook Of Research Synthesis.

[47] Egger M, Smith GD, Schneider M, Minder C (1997). Bias in meta-analysis detected by a simple, graphical test. Br Med J.

[48] Duval S, Tweedie R (2000). Trim and fill: a simple funnel-plot-based method of testing and adjusting for publication bias in meta-analysis. Biometrics.

[49] Tao D, Xie L, Wang T, Wang T (2015). A meta-analysis of the use of electronic reminders for patient adherence to medication in chronic disease care. J Telemed Telecare.

[50] Safren SA, Hendriksen ES, Desousa N, Boswell SL, Mayer KH (2003). Use of an on-line pager system to increase adherence to antiretroviral medications. AIDS Care.

[51] Reynolds NR, Testa MA, Su M, Chesney MA, Neidig JL, Frank I, Smith S, Ickovics J, Robbins GK, AIDS Clinical Trials Group 731384 Teams (2008). Telephone support to improve antiretroviral medication adherence: a multisite, randomized controlled trial. J Acquir Immune Defic Syndr.

[52] Simoni JM, Huh D, Frick PA, Pearson CR, Andrasik MP, Dunbar PJ, Hooton TM (2009). Peer support and pager messaging to promote antiretroviral modifying therapy in Seattle: a randomized controlled trial. J Acquir Immune Defic Syndr.

[53] Lester RT, Ritvo P, Mills EJ, Kariri A, Karanja S, Chung MH, Jack W, Habyarimana J, Sadatsafavi M, Najafzadeh M, Marra CA, Estambale B, Ngugi E, Ball TB, Thabane L, Gelmon LJ, Kimani J, Ackers M, Plummer FA (2010). Effects of a mobile phone short message service on antiretroviral treatment adherence in Kenya (WelTel Kenya1): a randomised trial. Lancet.

[54] Pop-Eleches C, Thirumurthy H, Habyarimana JP, Zivin JG, Goldstein MP, de Walque D, MacKeen L, Haberer J, Kimaiyo S, Sidle J, Ngare D, Bangsberg DR (2011). Mobile phone technologies improve adherence to antiretroviral treatment in a resource-limited setting: a randomized controlled trial of text message reminders. AIDS.

[55] Mbuagbaw L, Thabane L, Ongolo-Zogo P, Lester RT, Mills EJ, Smieja M, Dolovich L, Kouanfack C (2012). The Cameroon Mobile Phone SMS (CAMPS) trial: a randomized trial of text messaging versus usual care for adherence to antiretroviral therapy. PLoS One.

[56] da Costa TM, Barbosa BJ, Costa DA, Sigulem D, de Fátima MH, Filho AC, Pisa IT (2012). Results of a randomized controlled trial to assess the effects of a mobile SMS-based intervention on treatment adherence in HIV/AIDS-infected Brazilian women and impressions and satisfaction with respect to incoming messages. Int J Med Inform.

[57] Hersch RK, Cook RF, Billings DW, Kaplan S, Murray D, Safren S, Goforth J, Spencer J (2013). Test of a web-based program to improve adherence to HIV medications. AIDS Behav.

[58] Shet A, de Costa A, Kumarasamy N, Rodrigues R, Rewari BB, Ashorn P, Eriksson B, Diwan V, HIVIND Study Team (2014). Effect of mobile telephone reminders on treatment outcome in HIV: evidence from a randomised controlled trial in India. Br Med J.

[59] Robbins GK, Testa MA, Su M, Safren SA, Morse G, Lammert S, Shafer RW, Reynolds NR, Chesney MA (2013). Site nurse-initiated adherence and symptom support telephone calls for HIV-positive individuals starting antiretroviral therapy, ACTG 5031: substudy of ACTG 384. HIV Clin Trials.

[60] Sabin LL, DeSilva MB, Gill CJ, Zhong L, Vian T, Xie W, Cheng F, Xu K, Lan G, Haberer JE, Bangsberg DR, Li Y, Lu H, Gifford AL (2015). Improving adherence to antiretroviral therapy with triggered real-time text message reminders: the China adherence through technology study. J Acquir Immune Defic Syndr.

[61] Ingersoll KS, Dillingham RA, Hettema JE, Conaway M, Freeman J, Reynolds G, Hosseinbor S (2015). Pilot RCT of bidirectional text messaging for ART adherence among nonurban substance users with HIV. Health Psychol.

[62] Belzer ME, Naar-King S, Olson J, Sarr M, Thornton S, Kahana SY, Gaur AH, Clark LF, Adolescent Medicine Trials Network for HIV/AIDS Interventions (2014). The use of cell phone support for non-adherent HIV-infected youth and young adults: an initial randomized and controlled intervention trial. AIDS Behav.

[63] Orrell C, Cohen K, Mauff K, Bangsberg DR, Maartens G, Wood R (2015). A randomized controlled trial of real-time electronic adherence monitoring with text message dosing reminders in people starting first-line antiretroviral therapy. J Acquir Immune Defic Syndr.

[64] Garofalo R, Kuhns LM, Hotton A, Johnson A, Muldoon A, Rice D (2016). A randomized controlled trial of personalized text message reminders to promote medication adherence among HIV-positive adolescents and young adults. AIDS Behav.

[65] Ruan Y, Xiao X, Chen J, Li X, Williams AB, Wang H (2017). Acceptability and efficacy of interactive short message service intervention in improving HIV medication adherence in Chinese antiretroviral treatment-naïve individuals. Patient Prefer Adherence.

[66] Reid MJ, Steenhoff AP, Thompson J, Gabaitiri L, Cary MS, Steele K, Mayisela S, Dickinson D, Ehrenkranz P, Friedman HM, Linkin DR (2017). Evaluation of the effect of cellular SMS reminders on consistency of antiretroviral therapy pharmacy pickups in HIV-infected adults in Botswana: a randomized controlled trial. Health Psychol Behav Med.

[67] Abdulrahman SA, Rampal L, Ibrahim F, Radhakrishnan AP, Shahar HK, Othman N (2017). Mobile phone reminders and peer counseling improve adherence and treatment outcomes of patients on ART in Malaysia: a randomized clinical trial. PLoS One.

[68] Linnemayr S, Huang H, Luoto J, Kambugu A, Thirumurthy H, Haberer JE, Wagner G, Mukasa B (2017). Text messaging for improving antiretroviral therapy adherence: no effects after 1 year in a randomized controlled trial among adolescents and young adults. Am J Public Health.

[69] Conn VS, Ruppar TM (2017). Medication adherence outcomes of 771 intervention trials: systematic review and meta-analysis. Prev Med.

[70] Lowther K, Selman L, Harding R, Higginson IJ (2014). Experience of persistent psychological symptoms and perceived stigma among people with HIV on antiretroviral therapy (ART): a systematic review. Int J Nurs Stud.

[71] Shubber Z, Mills EJ, Nachega JB, Vreeman R, Freitas M, Bock P, Nsanzimana S, Penazzato M, Appolo T, Doherty M, Ford N (2016). Patient-reported barriers to adherence to antiretroviral therapy: a systematic review and meta-analysis. PLoS Med.

[72] Mohammed MA, Moles RJ, Chen TF (2016). Medication-related burden and patients' lived experience with medicine: a systematic review and metasynthesis of qualitative studies. BMJ Open.

[73] Sidebottom D, Ekström AM, Strömdahl S (2018). A systematic review of adherence to oral pre-exposure prophylaxis for HIV - how can we improve uptake and adherence?. BMC Infect Dis.

[74] Burch LS, Smith CJ, Anderson J, Sherr L, Rodger AJ, O'Connell R, Geretti AM, Gilson R, Fisher M, Elford J, Jones M, Collins S, Azad Y, Phillips AN, Speakman A, Johnson MA, Lampe FC (2016). Socioeconomic status and treatment outcomes for individuals with HIV on antiretroviral treatment in the UK: cross-sectional and longitudinal analyses. Lancet Public Health.

[75] Sterne JA, Egger M (2001). Funnel plots for detecting bias in meta-analysis: guidelines on choice of axis. J Clin Epidemiol.

[76] Kjaergard LL, Villumsen J, Gluud C (2001). Reported methodologic quality and discrepancies between large and small randomized trials in meta-analyses. Ann Intern Med.

[77] Zhang Z, Xu X, Ni H (2013). Small studies may overestimate the effect sizes in critical care meta-analyses: a meta-epidemiological study. Critical Care.

[78] Vervloet M, Linn AJ, van Weert JC, de Bakker DH, Bouvy ML, van Dijk L (2012). The effectiveness of interventions using electronic reminders to improve adherence to chronic medication: a systematic review of the literature. J Am Med Inform Assoc.

[79] Wald DS, Butt S, Bestwick JP (2015). One-way versus two-way text messaging on improving medication adherence: meta-analysis of randomized trials. Am J Med.

[80] Thaler RH, Sunstein CR (2008). Nudge: Improving Decisions About Health, Wealth, and Happiness.

[81] Hall MG, Marteau TM, Sunstein CR, Ribisl KM, Noar SM, Orlan EN, Brewer NT (2018). Public support for pictorial warnings on cigarette packs: an experimental study of US smokers. J Behav Med.

[82] Arno A, Thomas S (2016). The efficacy of nudge theory strategies in influencing adult dietary behaviour: a systematic review and meta-analysis. BMC Public Health.

[83] Harrison JD, Jones JM, Small DS, Rareshide CA, Szwartz G, Steier D, Guszcza J, Kalra P, Torio B, Reh G, Hilbert V, Patel MS (2019). Social incentives to encourage physical activity and understand predictors (STEP UP): design and rationale of a randomized trial among overweight and obese adults across the United States. Contemp Clin Trials.

[84] Lam WY, Fresco P (2015). Medication adherence measures: an overview. Biomed Res Int.

[85] Sangeda RZ, Mosha F, Prosperi M, Aboud S, Vercauteren J, Camacho RJ, Lyamuya EF, van Wijngaerden E, Vandamme A (2014). Pharmacy refill adherence outperforms self-reported methods in predicting HIV therapy outcome in resource-limited settings. BMC Public Health.

