# Supplementary material for: Electronic Health Interventions to Improve Adherence to Antiretroviral Therapy in People Living With HIV: Systematic Review and Meta-Analysis
Source: JMIR Mhealth Uhealth. 2019 Oct 16;7(10):e14404. doi: 10.2196/14404 (PMC6913542; doi:10.2196/14404)
Supplement: Multimedia Appendix 1 [file mhealth_v7i10e14404_app1.pdf]

Multimedia Appendix 1. Reviews literature search strategy

No	Terms
**#1**	HIV OR human immunodeficiency virus OR AIDS OR acquired immunodeficiency syndrome OR acquired immune deficiency syndrome OR acquired immuno-deficiency syndrome OR HIV positive OR HIV infection
**#2**	antiretroviral OR anti-retroviral OR antiretroviral therapy OR ART OR highly active antiretroviral therapy OR HAART OR Anti-HIV Agents
**#3**	adherence OR compliance OR medication adherence OR medication compliance OR Medication Nonadherence OR Medication Noncompliance OR Medication Persistence
**#4**	systematic review OR meta-analysis
**#5**	#1 AND #2 AND #3 AND #4
