# Supplementary material for: Electronic Health Interventions to Improve Adherence to Antiretroviral Therapy in People Living With HIV: Systematic Review and Meta-Analysis
Source: JMIR Mhealth Uhealth. 2019 Oct 16;7(10):e14404. doi: 10.2196/14404 (PMC6913542; doi:10.2196/14404)
Supplement: Multimedia Appendix 2 [file mhealth_v7i10e14404_app2.pdf]

Multimedia Appendix 2. Formal systematic literature search strategy

No	Terms
**#1**	HIV OR human immunodeficiency virus OR AIDS OR acquired immunodeficiency syndrome OR acquired immune deficiency syndrome OR acquired immuno-deficiency syndrome OR HIV positive OR HIV infection
**#2**	antiretroviral OR anti-retroviral OR antiretroviral therapy OR ART OR highly active antiretroviral therapy OR HAART OR Anti-HIV Agents
**#3**	adherence OR compliance OR medication adherence OR medication compliance OR Medication Nonadherence OR Medication Noncompliance OR Medication Persistence
**#4**	electronic health OR eHealth OR mHealth OR telemedicine OR mobile phone OR Cellular Phone OR SMS OR text message OR texting OR text messaging OR telephone OR phone OR Online OR social media OR social web OR social network OR social networking OR social software OR social medium OR social gaming OR geosocial networking OR chat room OR blogging OR webcast OR web 2.0 OR web2 OR medicine 2.0 OR health 2.0 OR blog OR podcast OR vodcast OR web log OR microblogging OR mobile app OR mobile application OR mobile health applications OR mobile HIV applications OR mobile technology OR Facebook OR YouTube OR twitter OR tweet OR Instagram OR LinkedIn OR Pinterest OR Reddit OR Second Life OR Tumblr OR Weibo OR WeChat OR QQ OR Qzone OR Skype OR Snapchat OR V Kontakte OR Whatsapp OR Baidu tieba OR Viber OR Messenger OR Tinder OR OkCupid OR LINE OR gay apps OR Grindr OR Scruff OR Growlr OR Blued OR Hornet OR Zank
**#5**	#1 AND #2 AND #3 AND #4
