# Supplementary material for: Electronic Health Interventions to Improve Adherence to Antiretroviral Therapy in People Living With HIV: Systematic Review and Meta-Analysis
Source: JMIR Mhealth Uhealth. 2019 Oct 16;7(10):e14404. doi: 10.2196/14404 (PMC6913542; doi:10.2196/14404)
Supplement: Multimedia Appendix 3 [file mhealth_v7i10e14404_app3.pdf]

Multimedia Appendix 3. Population, interventions, comparisons, outcomes and study design (PICOS) criteria for study inclusion

Criteria	Definition
**Population**	People living with HIV (PLWH) on antiretroviral therapy (ART)
**Interventions**	Any eHealth into 3 groups:<br>(1) Non-internet based eHealth: SMS (short message service) and phone calls only;<br>(2) Internet-based eHealth: social media, software, websites and mobile applications;<br>(3) Combination (included both (SMS/phone calls) and internet-based eHealth).<br>OR Usual standard of care plus any eHealth<br>to improve adherence to ART
**Comparisons**	Usual standard of care
**Outcomes**	Report at least one adherence measurement to ART<br>Adherence outcome (i.e. self-report, pill count, electronic monitoring, pharmacy refill record and treatment interruptions) OR biological outcome (i.e. Viral load (log10 copies/mL), CD4+ count and VS/VF (viral suppression or virological failure))
**Study Design**	Randomized Controlled Trial (RCT) with at least 3-month follow-up
