# Supplementary material for: Electronic Health Interventions to Improve Adherence to Antiretroviral Therapy in People Living With HIV: Systematic Review and Meta-Analysis
Source: JMIR Mhealth Uhealth. 2019 Oct 16;7(10):e14404. doi: 10.2196/14404 (PMC6913542; doi:10.2196/14404)
Supplement: Multimedia Appendix 4 [file mhealth_v7i10e14404_app4.pdf]

Multimedia Appendix 4. Cochrane risk of bias quality assessment for included studies

Study	Random sequence generation	Allocation concealment	Blinding of participants and personnel	Blinding of outcome assessment	Incomplete outcome data	Selective outcome reporting	Other sources of bias	Comments	Overall study quality
Safren et al., 2003	Unclear risk	Low risk	High risk	High risk	High risk	Low risk	Low risk		low
ACTG 731 study team., 2008	Unclear risk	Low risk	High risk	High risk	High risk	Low risk	Low risk		low
Simoni et al., 2009	Low risk	Low risk	Unclear risk	Unclear risk	Low risk	Low risk	Low risk		high
WelTel Kenya1 study team., 2010	Low risk	Low risk	Low risk	Low risk	Low risk	Low risk	Low risk		high
Pop-Eleches et al., 2011	Low risk	Low risk	High risk	High risk	Low risk	Low risk	Low risk		high
CAMPS study team., 2012	Low risk	Low risk	Low risk	Low risk	Low risk	Low risk	Low risk		high
da Costa et al., 2012	Low risk	Low risk	Low risk	Low risk	High risk	Low risk	High risk	Very small sample size	high
Hersch et al., 2013	Low risk	Low risk	Low risk	Low risk	High risk	Low risk	Low risk		high
HIVIND study team., 2014	Low risk	Low risk	High risk	High risk	Low risk	Low risk	Low risk		high
ACTG 5031 study team., 2014	Low risk	Low risk	Unclear risk	Unclear risk	Low risk	Low risk	Low risk		high
Sabin et al., 2015	Low risk	Low risk	High risk	Low risk	Low risk	Low risk	Low risk		high

Ingersoll et al., 2015	Unclear risk	Low risk	High risk	High risk	Low risk	Low risk	Low risk		low
Belzer et al., 2015	Low risk	Low risk	Unclear risk	Unclear risk	High risk	Low risk	High risk	Very small sample size	high
Orrell et al., 2015	Unclear risk	Low risk	High risk	High risk	Unclear risk	Low risk	Low risk		low
Garofalo et al., 2016	Low risk	Low risk	High risk	High risk	High risk	Low risk	Unclear risk		high
Ruan et al., 2017	Low risk	Low risk	Unclear risk	Unclear risk	Low risk	High risk	Low risk		high
Reid et al., 2017	Unclear risk	Low risk	Low risk	Low risk	Low risk	High risk	Low risk		high
Abdulrahman et al., 2017	Low risk	Low risk	Low risk	Low risk	Low risk	Low risk	Low risk		high
Linnemayr et al., 2017	Low risk	Low risk	Low risk	Low risk	Low risk	Low risk	Low risk		high
