# Supplementary material for: Electronic Health Interventions to Improve Adherence to Antiretroviral Therapy in People Living With HIV: Systematic Review and Meta-Analysis
Source: JMIR Mhealth Uhealth. 2019 Oct 16;7(10):e14404. doi: 10.2196/14404 (PMC6913542; doi:10.2196/14404)
Supplement: Multimedia Appendix 5 [file mhealth_v7i10e14404_app5.pdf]

Multimedia Appendix 5. List of included studies after full-text review

Study	Author	Year	Title
Safren et al.	Safren et al.	2003	**Use of an on-line pager system to increase adherence to antiretroviral medications**
ACTG 731 study team.	Reynolds et al.	2008	**Telephone support to improve antiretroviral medication adherence: a multisite, randomized controlled trial**
Simoni et al.	Simoni et al.	2009	**Peer support and pager messaging to promote antiretroviral modifying therapy in Seattle: a randomized controlled trial**
WelTel Kenya1 study team.	Lester et al.	2010	**Effects of a mobile phone short message service on antiretroviral treatment adherence in Kenya (WelTel Kenya1): A randomised trial**
Pop-Eleches et al.	Pop-Eleches et al.	2011	**Mobile phone technologies improve adherence to antiretroviral treatment in a resource-limited setting: A randomized controlled trial of text message reminders**
CAMPS study team.	Mbuagbaw et al.	2012	**The Cameroon Mobile Phone SMS (CAMPS) Trial: A Randomized Trial of Text Messaging versus Usual Care for Adherence to Antiretroviral Therapy**
da Costa et al.	da Costa et al.	2012	**Results of a randomized controlled trial to assess the effects of a mobile SMS-based intervention on treatment adherence in HIV/AIDS-infected Brazilian women and impressions and satisfaction with respect to incoming messages**
Hersch et al.	Hersch et al.	2013	**Test of a web-based program to improve adherence to HIV medications**
HIVIND study team.	Shet et al.	2014	**Effect of mobile telephone reminders on treatment outcome in HIV: Evidence from a randomised controlled trial in India**
ACTG 5031	Robbins et al.	2014	**Site nurse-initiated adherence and symptom support**

study team.			**telephone calls for HIV-positive individuals starting antiretroviral therapy, ACTG 5031: substudy of ACTG 384**
Sabin et al.	Sabin et al.	2015	**Improving Adherence to Antiretroviral Therapy with Triggered Real-time Text Message Reminders: The China Adherence Through Technology Study**
Ingersoll et al.	Ingersoll et al.	2015	**Pilot RCT of bidirectional text messaging for ART adherence among nonurban substance users with HIV**
Belzer et al.	Belzer et al.	2015	**The use of cell phone support for non-adherent HIV-infected youth and young adults: an initial randomized and controlled intervention trial**
Orrell et al.	Orrell et al.	2015	**A Randomized Controlled Trial of Real-Time Electronic Adherence Monitoring With Text Message Dosing Reminders in People Starting First-Line Antiretroviral Therapy**
Garofalo et al.	Garofalo et al.	2016	**A Randomized Controlled Trial of Personalized Text Message Reminders to Promote Medication Adherence Among HIV-Positive Adolescents and Young Adults**
Ruan et al.	Ruan et al.	2017	**Acceptability and efficacy of interactive short message service intervention in improving HIV medication adherence in Chinese antiretroviral treatment-naive individuals**
Reid et al.	Reid et al.	2017	**Evaluation of the effect of cellular SMS reminders on consistency of antiretroviral therapy pharmacy pickups in HIV-infected adults in Botswana: a randomized controlled trial**
Abdulrahman et al.	Abdulrahman et al.	2017	**Mobile phone reminders and peer counseling improve adherence and treatment outcomes of patients on ART in Malaysia: A randomized clinical trial**

Linnemayr et al.	Linnemayr et al.	2017	**Text Messaging for Improving Antiretroviral Therapy Adherence: no Effects After 1 Year in a Randomized Controlled Trial Among Adolescents and Young Adults**
