# Supplementary material for: Electronic Health Interventions to Improve Adherence to Antiretroviral Therapy in People Living With HIV: Systematic Review and Meta-Analysis
Source: JMIR Mhealth Uhealth. 2019 Oct 16;7(10):e14404. doi: 10.2196/14404 (PMC6913542; doi:10.2196/14404)
Supplement: Multimedia Appendix 6 [file mhealth_v7i10e14404_app6.pdf]

Multimedia Appendix 6. List of excluded studies after full-text review

Author	Title	Year	Reason
Carol et al.	A 2-arm, randomized, controlled trial of a motivational interviewing-based intervention to improve adherence to antiretroviral therapy (ART) among patients failing or initiating ART	2006	Intervention
Belzer et al.	Acceptability and Feasibility of a Cell Phone Support Intervention for Youth Living with HIV with Nonadherence to Antiretroviral Therapy	2015	Study design
Krummenacher et al.	Antiretroviral adherence program in HIV patients: A feasibility study in the Swiss HIV Cohort Study	2010	Study design
Saberi et al.	Antiretroviral Therapy Adherence and Use of an Electronic Shared Medical Record Among People Living with HIV	2016	Intervention
Taiwo et al.	Assessing the viorologic and adherence benefits of patient-selected HIV treatment partners in a resource-limited setting	2010	Intervention
Raymond et al.	Association Between the Occurrence of Adverse Drug Events and Modification of First-Line Highly Active Antiretroviral Therapy in Ghanaian HIV Patients	2016	Intervention
Finocchario-Kessler et al.	Baseline predictors of ninety percent or higher antiretroviral therapy adherence in a diverse urban sample: the role of patient autonomy and fatalistic religious beliefs	2011	Intervention
Gwadz et al.	Behavioral intervention improves treatment outcomes among HIV-infected individuals who have delayed, declined, or discontinued antiretroviral therapy: a randomized controlled trial of a novel	2016	Intervention

	intervention		
Evans et al.	Can Short-Term Use of Electronic Patient Adherence Monitoring Devices Improve Adherence in Patients Failing Second-Line Antiretroviral Therapy? Evidence from a Pilot Study in Johannesburg, South Africa	2016	Study design
McMahon et al.	Clinic Network Collaboration and Patient Tracing to Maximize Retention in HIV Care	2015	Comparators
Cook et al.	Cognitive functioning, depression, and HIV medication adherence in India: a randomized pilot trial	2014	Intervention
Elul et al.	A combination intervention strategy to improve linkage to and retention in HIV care following diagnosis in Mozambique: a cluster-randomized study	2017	Outcomes
Siedner et al.	A combination SMS and transportation reimbursement intervention to improve HIV care following abnormal CD4 test results in rural Uganda: a prospective observational cohort study	2015	Comparators
Batya et al.	A combination strategy for enhancing linkage to and retention in HIV care among adults newly diagnosed with HIV in Mozambique: study protocol for a site-randomized implementation science study	2014	Protocol
Delp et al.	Communicating information to patients: the use of cartoon illustrations to improve comprehension of instructions	1996	Intervention
Megan et al.	Community-Based Accompaniment with Supervised Antiretrovirals for HIV-Positive Adults in Peru: A Cluster-Randomized Trial	2018	Intervention

Martin et al.	Computer-generated reminders and quality of pediatric HIV care in a resource-limited setting	2013	Study design
Remien et al.	Couple-focused support to improve HIV medication adherence: a randomized controlled trial	2005	Intervention
Williams et al.	Cultural adaptation of an evidence-based nursing intervention to improve medication adherence among people living with HIV/AIDS (PLWHA) in China	2013	Intervention
Deribe et al.	Defaulters from antiretroviral treatment in Jimma University Specialized Hospital, Southwest Ethiopia	2008	Study design
Jones et al.	Determinants of engagement in HIV treatment and care among Zambians new to antiretroviral therapy	2013	Intervention
Glasner et al.	Development of a cognitive behavioral therapy-based text messaging intervention for HIV-infected alcohol users	2017	Conference Abstract
Luft et al.	Distance and geographic factors on HIV treatment outcomes in a kenyan cohort of HIV patients enrolled in an SMS program (WelTel Kenya1)	2013	Conference Abstract
Ankrah et al.	The effect of adherence on treatment change among HIV/AIDS patients at the korle-bu teaching hospital in Ghana	2013	Conference Abstract
Awiti et al.	The effect of an interactive weekly mobile phone messaging on retention in prevention of mother to child transmission (PMTCT) of HIV program: study protocol for a randomized controlled trial (WELTEL PMTCT)	2016	Protocol
Lubega et al.	Effect of Community Support Agents on Retention of People Living With HIV in Pre-antiretroviral Care: a Randomized Controlled Trial in Eastern	2015	Intervention

	Uganda		
Nakimuli-Mpungu et al.	The Effect of Group Support Psychotherapy Delivered by Trained Lay Health Workers for Depression Treatment Among People with HIV in Uganda: Protocol of a Pragmatic, Cluster Randomized Trial	2017	Protocol
Cantudo-Cuenca et al.	Effect of mobile phone short messages on antiretroviral treatment adherence among HIV infected patients	2016	Conference Abstract
Chang et al.	Effect of peer health workers on AIDS care in Rakai, Uganda: a cluster-randomized trial	2010	Participants
Coleman et al.	Effectiveness of an SMS-based maternal mHealth intervention to improve clinical outcomes of HIV-positive pregnant women	2017	Outcomes
Pyne et al.	Effectiveness of collaborative care for depression in human immunodeficiency virus clinics	2011	Intervention
Tam et al.	Effectiveness of HIV self-management support group program for aboriginal and non-aboriginal peoples living in Vancouver's downtown eastside	2011	Conference Abstract
Konkle-Parker et al.	Effects of an intervention addressing information, motivation, and behavioral skills on HIV care adherence in a southern clinic cohort	2014	Intervention
Wang et al.	Effects of nurse-delivered home visits combined with telephone calls on medication adherence and quality of life in HIV-infected heroin users in Hunan of China	2010	Intervention
Huis et al.	The efficacy of a brief intervention to reduce alcohol misuse in patients with HIV in South Africa: study protocol for a randomized controlled trial	2012	Protocol

Peltzer et al.	Efficacy of a lay health worker led group antiretroviral medication adherence training among non-adherent HIV-positive patients in KwaZulu-Natal, South Africa: results from a randomized trial	2012	Intervention
Wyatt et al.	The efficacy of an integrated risk reduction intervention for HIV-positive women with child sexual abuse histories	2004	Intervention
Uzma et al.	Efficacy of interventions for improving antiretroviral therapy adherence in HIV/AIDS cases at PIMS, Islamabad	2011	Study design
de Bruin et al.	Electronic monitoring-based counseling to enhance adherence among HIV-infected patients: a randomized controlled trial	2010	Intervention
Jones et al.	Enhancing HIV medication adherence in India	2013	Study design
Reuben et al.	Enhancing Lay Counselor Capacity to Improve Patient Outcomes with Multimedia Technology	2015	Intervention
Mavhu et al.	Evaluating a multi-component, community-based program to improve adherence and retention in care among adolescents living with HIV in Zimbabwe: Study protocol for a cluster randomized controlled trial	2017	Protocol
Sando et al.	Evaluation of a community health worker intervention and the World Health Organization's Option B versus Option A to improve antenatal care and PMTCT outcomes in Dar es Salaam, Tanzania: study protocol for a cluster-randomized controlled health systems implementation trial	2014	Protocol
Côté et al.	Evaluation of a real-time virtual intervention to empower persons living with HIV to use therapy	2012	Protocol

	self-management: study protocol for an online randomized controlled trial		
Drake et al.	Evaluation of mHealth strategies to optimize adherence and efficacy of Option B+ prevention of mother-to-child HIV transmission: Rationale, design and methods of a 3-armed randomized controlled trial.	2017	Protocol
Basso et al.	Exploring ART intake scenes in a human rights-based intervention to improve adherence: a randomized controlled trial	2013	Intervention
Masquillier et al.	Families as catalysts for peer adherence support in enhancing hope for people living with HIV/AIDS in South Africa	2014	Outcomes
Sutton et al.	Feasibility and Acceptability of Health Communication Interventions Within a Combination Intervention Strategy for Improving Linkage and Retention in HIV Care in Mozambique	2017	Outcomes
Petersen et al.	A group-based counselling intervention for depression comorbid with HIV/AIDS using a task shifting approach in South Africa: a randomized controlled pilot study	2014	Intervention
Lester et al.	The HAART cell phone adherence trial (WelTel Kenya1): A randomized controlled trial protocol	2009	Protocol
Bodenhamer et al.	Hiv self-management support for aboriginal and non-aboriginal peoples living in vancouver's downtown eastside - The impact on antiretroviral adherence and uptake	2010	Conference Abstract
Hayes et al.	HPTN 071 (PopART): rationale and design of a cluster-randomised trial of the population impact of	2014	Protocol

	an HIV combination prevention intervention including universal testing and treatment - a study protocol for a cluster randomised trial		
Nyamathi et al.	Impact of a rural village women (Asha) intervention on adherence to antiretroviral therapy in southern India	2012	Intervention
Byrd-Glover et al.	Impact of a specialized telephone medication therapy consultation program on the adherence to antiretroviral therapy among human immunodeficiency virus-positive adults	2016	Conference Abstract
Rathbun et al.	Impact of an adherence clinic on behavioral outcomes and virologic response in treatment of HIV infection: a prospective, randomized, controlled pilot study	2005	Intervention
Walsh et al.	Impact of early initiation versus national standard of care of antiretroviral therapy in Swaziland's public sector health system: study protocol for a stepped-wedge randomized trial	2017	Protocol
Gross et al.	Impact of managed problem solving (MAPS) antiretroviral adherence intervention on depressive symptoms	2014	Intervention
Nakigozi et al.	Impact of Patient-Selected Care Buddies on Adherence to HIV Care, Disease Progression, and Conduct of Daily Life Among Pre-antiretroviral HIV-Infected Patients in Rakai, Uganda: A Randomized Controlled Trial	2015	Intervention
Cuong et al.	Impact of peer support on virologic failure in HIV-infected patients on antiretroviral therapy - a cluster randomized controlled trial in Vietnam	2016	Intervention

Nyamathi et al.	Impact of protein supplementation and care and support on body composition and CD4 count among HIV-infected women living in rural India: results from a randomized pilot clinical trial	2013	Intervention
Kunutsor et al.	Improving clinic attendance and adherence to antiretroviral therapy through a treatment supporter intervention in Uganda: a randomized controlled trial	2011	Intervention
Johnson et al.	Improving coping skills for self-management of treatment side effects can reduce antiretroviral medication nonadherence among people living with HIV	2011	Intervention
van Loggerenberg et al.	Individualised motivational counselling to enhance adherence to antiretroviral therapy is not superior to didactic counselling in South African patients: findings of the CAPRISA 058 randomised controlled trial	2015	Comparators
Moore et al.	Individualized texting for adherence building (iTAB) improves cart adherence in HIV-infected persons with co-occurring bipolar disorder	2015	Comparators
Moore et al.	Individualized texting for adherence building (iTAB): improving antiretroviral dose timing among HIV-infected persons with co-occurring bipolar disorder	2015	Comparators
Jones et al.	Influencing medication adherence among women with AIDS	2003	Study design
Kurth et al.	Linguistic and Cultural Adaptation of a Computer-Based Counseling Program (CARE+ Spanish) to Support HIV Treatment Adherence and Risk Reduction for People Living With HIV/AIDS: A	2016	Intervention

	Randomized Controlled Trial		
McNairy et al.	The Link4Health study to evaluate the effectiveness of a combination intervention strategy for linkage to and retention in HIV care in Swaziland: protocol for a cluster randomized trial	2015	Protocol
Holstad et al.	The LIVE Network: a music-based messaging program to promote ART adherence self-management	2013	Intervention
Dexter et al.	A Longitudinal Analysis of Antiretroviral Adherence Among Young Black Men Who Have Sex With Men	2017	Intervention
Reynolds et al.	MAHILA: a protocol for evaluating a nurse-delivered mHealth intervention for women with HIV and psychosocial risk factors in India	2016	Protocol
Odeny et al.	Maximizing adherence and retention for women living with HIV and their infants in Kenya (MOTIVATE! study): Study protocol for a randomized controlled trial	2018	Protocol
Smith et al.	A medication self-management program to improve adherence to HIV therapy regimens	2003	Intervention
Kalichman et al.	Mobile Health Intervention to Reduce HIV Transmission: A Randomized Trial of Behaviorally Enhanced HIV Treatment as Prevention (B-TasP)	2018	Comparators
Schnall et al.	Mobile Health Technology for Improving Symptom Management in Low Income Persons Living with HIV	2018	Outcomes
Elizabeth et al.	Mobile Text Messaging to Improve Medication Adherence and Viral Load in a Vulnerable Canadian Population Living With Human Immunodeficiency Virus: A Repeated Measures Study	2017	Comparators

Kalichman et al.	Monthly unannounced pill counts for monitoring HIV treatment adherence: Tests for self-monitoring and reactivity effects	2010	Comparators
Naar-King et al.	Motivational Enhancement System for Adherence (MESA): pilot randomized trial of a brief computer-delivered prevention intervention for youth initiating antiretroviral treatment	2013	Intervention
León et al.	A new multidisciplinary home care telemedicine system to monitor stable chronic human immunodeficiency virus-infected patients: a randomized study	2011	Intervention
Mbuagbaw et al.	Opening communication channels with people living with HIV using mobile phone text messaging: insights from the CAMPS trial	2013	Comparators
Mitchell et al.	Participants' descriptions of social support within a multisite intervention for HIV-seropositive injection drug users (INSPIRE)	2007	Study design
Gross et al.	Partner-based adherence intervention for second-line antiretroviral therapy (ACTG A5234): a multinational randomised trial	2015	Intervention
Gross et al.	Partner-Based Intervention for Adherence To Second-Line ART: a Multinational Trial (ACTG A5234)	2015	Intervention
Gross et al.	Partner-Focused Adherence Intervention for Second-line Antiretroviral Therapy: A Multinational Randomized Trial (ACTG A5234)	2015	Intervention
Finocchario-Kessler et al.	Patient communication tools to enhance ART adherence counseling in low and high resource settings	2012	Outcomes

Salmoirago-Blotcher et al.	Phone-delivered mindfulness training to promote medication adherence and reduce sexual risk behavior among persons living with HIV: Design and methods	2017	Intervention
Himelhoch et al.	Pilot feasibility study of Heart2HAART: a smartphone application to assist with adherence among substance users living with HIV	2017	Intervention
Claborn et al.	Pilot study examining the efficacy of an electronic intervention to promote HIV medication adherence	2014	Study design
McCoy et al.	Pilot study of a multi-pronged intervention using social norms and priming to improve adherence to antiretroviral therapy and retention in care among adults living with HIV in Tanzania	2017	Intervention
Crouch et al.	A pilot study to evaluate the magnitude of association of the use of electronic personal health records with patient activation and empowerment in HIV-infected veterans	2015	Study design
Belzer et al.	A pilot study using cell phone interactions to improve HIV medication adherence in adolescents who have previously failed antiretroviral therapy	2013	Conference Abstract
Moore et al.	Preliminary Evidence for Feasibility, Use, and Acceptability of Individualized Texting for Adherence Building for Antiretroviral Adherence and Substance Use Assessment among HIV-Infected Methamphetamine Users	2013	Study design
Chiou et al.	A programme of symptom management for improving quality of life and drug adherence in AIDS/HIV patients	2006	Study design
Wagner et al.	Protocol for a Randomized Controlled Trial	2016	Protocol

	Evaluating Mobile Text Messaging to Promote Retention and Adherence to Antiretroviral Therapy for People Living With HIV in Burkina Faso		
Vaccher et al.	Protocol for an open-label, single-arm trial of HIV pre-exposure prophylaxis (PrEP) among people at high risk of HIV infection: the NSW Demonstration Project PRELUDE	2016	Protocol
Hickey et al.	Pulling the network together: Quasiexperimental trial of a patient-defined support network intervention for promoting engagement in HIV care and medication adherence on mfangano Island, Kenya	2015	Study design
Orrell et al.	Randomised controlled trial of text-message dosing reminders in patients starting ART	2015	Conference Abstract
Bigna et al.	A randomized blinded controlled trial of mobile phone reminders on the follow-up medical care of HIV-exposed and HIV-infected children in Cameroon: study protocol (MORE CARE)	2013	Protocol
Simoni et al.	A randomized controlled trial of a peer support intervention targeting antiretroviral medication adherence and depressive symptomatology in HIV-positive men and women	2007	Intervention
Hardy et al.	Randomized controlled trial of a personalized cellular phone reminder system to enhance adherence to antiretroviral therapy	2011	Comparators
Wohl et al.	Randomized controlled trial of an intervention to maintain suppression of HIV viremia after prison release: The impact trial	2017	Participants
Koenig et al.	Randomized controlled trial of an intervention to prevent adherence failure among HIV-infected	2008	Comparators

	patients initiating antiretroviral therapy		
Proeschold-Bell et al.	A randomized controlled trial of health information exchange between human immunodeficiency virus institutions	2010	Study design
Kalichman et al.	Randomized Factorial Trial of Phone-Delivered Support Counseling and Daily Text Message Reminders for HIV Treatment Adherence	2016	Comparators
Collier et al.	A randomized study of serial telephone call support to increase adherence and thereby improve virologic outcome in persons initiating antiretroviral therapy	2005	Outcomes
Horvath et al.	Results of an online social support andtext reminder ART intervention	2012	Conference Abstract
Vian et al.	The role of motivation in predicting antiretroviral therapy adherence in China	2016	Study design
L'Engle et al.	Scaled-Up Mobile Phone Intervention for HIV Care and Treatment: Protocol for a Facility Randomized Controlled Trial	2015	Protocol
Joseph et al.	SMSaude: Evaluating Mobile Phone Text Reminders to Improve Retention in HIV Care for Patients on Antiretroviral Therapy in Mozambique	2016	Outcomes
Wagner et al.	Supporting Treatment Adherence Readiness through Training (START) for patients with HIV on antiretroviral therapy: study protocol for a randomized controlled trial	2016	Protocol
Christopoulos et al.	A text messaging intervention to improve retention in care and virologic suppression in a U.S. urban safety-net HIV clinic: study protocol for the Connect4Care (C4C) randomized controlled trial	2014	Protocol
Jeffries et al.	Ucare4life: mobile texting to improve HIV care	2016	Conference

	continuum outcomes for minority youth		Abstract
Atukunda et al.	Understanding Patterns of Social Support and Their Relationship to an ART Adherence Intervention Among Adults in Rural Southwestern Uganda	2017	Outcomes
Sabin et al.	Using electronic drug monitor feedback to improve adherence to antiretroviral therapy among HIV-positive patients in China	2010	Intervention
Sorensen et al.	Voucher reinforcement improves medication adherence in HIV-positive methadone patients: a randomized trial	2007	Study design
Maduka et al.	Adherence counseling and reminder text messages improve uptake of antiretroviral therapy in a tertiary hospital in Nigeria	2013	Intervention
Kalichman et al.	Brief behavioral self-regulation counseling for HIV treatment adherence delivered by cell phone: An initial test of concept trial	2011	Comparators
Mbuagbaw et al.	The Cameroon mobile phone SMS (CAMPS) trial: a protocol for a randomized controlled trial of mobile phone text messaging versus usual care for improving adherence to highly active anti-retroviral therapy	2011	Protocol
Mbuagbaw et al.	The cameroon mobile phone SMS (CAMPS) trial: a randomized trial of text messaging versus usual care for adherence to antiretroviral therapy	2012	Conference Abstract
Thabane et al.	The cameroon mobile phone sms (CAMPS) trial: The protocol for a randomized controlled trial of mobile phone text messaging versus usual care for improving adherence to haart	2011	Conference Abstract
Suzette et al.	A Cognitive Behavioral Therapy-Based Text	2016	Protocol

	Messaging Intervention Versus Medical Management for HIV-Infected Substance Users: Study Protocol for a Pilot Randomized Trial		
Kurth et al.	Computerized counseling reduces HIV-1 viral load and sexual transmission risk: findings from a randomized controlled trial	2014	Outcomes
Costa et al.	Design of a randomized trial to evaluate the influence of mobile phone reminders on adherence to first line antiretroviral treatment in South India--the HIVIND study protocol	2010	Protocol
Brian et al.	The effect of antidepressant treatment on HIV and depression outcomes: Results from a randomized trial	2015	Study design
Mia et al.	The effect of weekly short message service communication on patient retention in care in the first year after HIV diagnosis: study protocol for a randomised controlled trial (WelTel Retain)	2013	Protocol
Margaret et al.	Effectiveness of a combination strategy for linkage and retention in adult HIV care in Swaziland: the Link4Health cluster randomized trial	2017	Intervention
Huang et al.	Effects of a Phone Call Intervention to Promote Adherence to Antiretroviral Therapy and Quality of Life of HIV/AIDS Patients in Baoshan, China: A Randomized Controlled Trial	2013	Outcomes
Williams et al.	Efficacy of an evidence-based ARV adherence intervention in China	2014	Intervention
Lippman et al.	Evaluation of short message service and peer navigation to improve engagement in HIV care in South Africa: Study protocol for a three-arm cluster	2016	Protocol

	randomized controlled trial		
Dowshen et al.	Evaluation of the feasibility and validity of short message system (SMS) text messaging for assessment of antiretroviral therapy adherence among youth living with HIV/AIDS (YLH)	2012	Conference Abstract
Horvath et al.	Feasibility, acceptability and preliminary efficacy of an online peer-to-peer social support ART adherence intervention	2013	Study design
Manzano et al.	Mobile phone text messaging to improve adherence to antiretroviral treatment in hiv-infected patients	2015	Conference Abstract
Nsagha et al.	A Randomized Controlled Trial on the Usefulness of Mobile Text Phone Messages to Improve the Quality of Care of HIV and AIDS Patients in Cameroon	2016	Outcomes
Haberer et al.	Short message service (SMS) reminders and real-time adherence monitoring improve antiretroviral therapy adherence in rural Uganda	2016	Study design
Perera et al.	Effect of a smartphone application incorporating personalized health-related imagery on adherence to antiretroviral therapy: a randomized clinical trial	2014	Comparators
