# Supplementary material for: Electronic Health Interventions to Improve Adherence to Antiretroviral Therapy in People Living With HIV: Systematic Review and Meta-Analysis
Source: JMIR Mhealth Uhealth. 2019 Oct 16;7(10):e14404. doi: 10.2196/14404 (PMC6913542; doi:10.2196/14404)
Supplement: Multimedia Appendix 7 [file mhealth_v7i10e14404_app7.pdf]

Multimedia Appendix 7. Study and participants characteristics of trials for principal systematic literature review

Trial	Country	Setting	Study duration	Intervention arm/ Control arm(IA / CA)	Sample Size (IA / CA)	Mean Age (Mean(SD))	Female ratio	Participant characteristics
**Safren et al., 2003**	USA	community healh center	12 weeks	Web-based computer program vs SOC	30 / 30	Not Reported	22%	90% or lower adherence after 2 week EDM surveillance
**ACTG 731 study team., 2008**	USA	University clinical trial group	64 weeks	Telephone calls vs SOC	54 / 55	36.4 for IA and 37.2 for CA	15%	HIV-1–infected individuals with ＜7 days of previous ART and no acute illnesses or opportunistic infections
**Simoni et al., 2009**	USA	HIV primary care outpatient clinic	9 months	SMS vs SOC	56 / 57	40 (8.2)	24%	Initiating or changing at least two medications of a HAART regimen
**WelTel Kenya1 study team., 2010**	Kenya	HIV clinic	12 months	SMS vs SOC	273 / 265	36.7 (8.5) for IA and 36.6 (7.9) for CA	65% for IA and 66% for CA	Initiated ART for the first time
**Pop-Eleches et al., 2011 (1)**	Kenya	HIV clinic	48 weeks	SMS vs SOC	142 / 69	35.7 for IA and 35.7 for CA	67%	Initiated ART <3 months
**Pop-Eleches et**	Kenya	HIV clinic	48 weeks	SMS vs SOC	147 / 70	37.3 for IA and	66%	Initiated ART <3 months

**al., 2011 (2)**						35.7 for CA		
**CAMPS study team., 2012**	Cameroon	Hospital clinic	6 months	SMS vs SOC	101 / 99	41.3 (10.1) for IA and 39.0 (10.0) for CA	68% for IA and 79% for CA	On ART regimen ⩾1 months
**da Costa et al., 2012**	Brazil	University clinic	4 months	SMS vs SOC	8 / 13	36.1 (9.1) for IA and 33.7 (5.3) for CA	100% for IA and 100% for CA	Stable patients with HIV
**Hersch et al., 2013**	USA	Large urban health clinic	9 months	Web-based computer programe vs SOC	79 / 89	46	27%	HIV+ on ART, not actively engaged in the clinic's medication adherence case management program for ≥ 6 months, viral load ＞48, and not diagnosed with any mental disorder
**HIVIND study team., 2014**	India	2 ambulatory clinics and 1 private HIV healthcare clinic	96 weeks	Telephone calls vs SOC	315 / 316	36.5 for IA and 37.2 for CA	43% for IA and 43% for CA	HIV+ ART naïve
**ACTG 5031 study team.,**	USA/Italy	AIDS Clinical Trial Site	64 weeks	Telephone calls vs SOC	166 / 167	36 for IA and 36 for CA	22% for IA and 19% for CA	HIV+, HIV RNA ≥ 500 copies/mL, ＜7 days of prior antiretroviral therapy, no serious acute

**2014**								illnesses or laboratory abnormality within 14 days of entry
**Sabin et al., 2015**	China	HIV clinic	9 months	EAMD vs SOC	63 / 56	36.9 (11.1) for IA and 38.4 (9.6) for CA	58% for IA and 66% for CA	receiving or initiating ART, deemed at risk for poor adherence by clinicians or themselves
**Ingersoll et al., 2015**	USA	primary HIV care clinics	6 months	SMS vs SOC	33 / 30	42.1 (9.1) for IA and 42.7 (11.0) for CA	39% for IA and 33% for CA	patients on ART, reported less than 95% ART adherence in the past 2 weeks, and used illicit drugs and/or drank at levels considered risky in the past 30 days
**Belzer et al., 2015**	USA	5 adolescent trials network sites	48 weeks	Telephone calls vs SOC	19 / 18	19.8 (2.5) for IA and 21.1 (2.5) for CA	42 % for IA and 33% for CA	HIV+ with a history of non-adherence to one or more components of ART: (a) currently prescribed ART and reporting to care provider adherence <90% and VL greater than 1,000 copies/ml (b) discontinued ART in the past while documented <90% adherent to last regimen, or (c) agreed to start ART but never initiated.

**Orrell et al., 2015**	South Africa	urban ART outpatient clinic	48 weeks	EAMD vs SOC	115 / 115	34.6 (9.2) for IA and 34.3 (9.0) for CA	64% for IA and 67% for CA	HIV+ ART naïve
**Garofalo et al., 2016**	USA	community-based health centers and other organizations using flyers and palm cards	6 months	SMS vs SOC	51 / 54	24.1 (3.2) for IA and 24.1 (2.7) for CA	20% for IA and 15% for CA	HIV+ on ART for ≥ 1 month with adherence problems (missed 1 dose in the past week or ≥4 doses in the last month)
**Ruan et al., 2017**	China	HIV clinic	6 months	SMS vs SOC	50 / 50	38.9 (9.8) for IA and 41.8 (9.8) for CA	38% for IA and 44% for CA	HIV+ on ART for no more than 3 months
**Reid et al., 2017**	Botswan a	urban private clinic	6 months	SMS vs SOC	54 / 54	40.8 for IA and 41.4 for CA	43% for IA and 46% for CA	HIV+ on ART
**Abdulrahman et al., 2017**	Malaysia	A semiurban based,	24 weeks	SMS + Telephone calls vs SOC	121 / 121	32.1 (8.7) for IA and 34.7	12% for IA and 11% for CA	HIV+ with completed four weeks of vitamin training and were newly initiating ART based

		Government-owned referral hospital				(9.5) for CA		on 2013 WHO guidelines
**Linnemayr et al., 2017 (1)**	Uganda	2 HIV care clinics	48 weeks	SMS vs SOC	110 / 56	18.5 for IA and 18.2 for CA	63% for IA and 57% for CA	HIV+ on ART or cotrimoxazole prophylaxis against common opportunistic infections
**Linnemayr et al., 2017 (2)**	Uganda	2 HIV care clinics	48 weeks	SMS vs SOC	110 / 56	18.3 for IA and 18.2 for CA	61% for IA and 57% for CA	HIV+ on ART or cotrimoxazole prophylaxis against common opportunistic infections
