# Supplementary material for: Electronic Health Interventions to Improve Adherence to Antiretroviral Therapy in People Living With HIV: Systematic Review and Meta-Analysis
Source: JMIR Mhealth Uhealth. 2019 Oct 16;7(10):e14404. doi: 10.2196/14404 (PMC6913542; doi:10.2196/14404)
Supplement: Multimedia Appendix 8 [file mhealth_v7i10e14404_app8.pdf]

Multimedia Appendix 8. Characteristics of eHealth intervention and outcome measures of trials for principal systematic literature review

Trial	Frequency of intervention	Intervention content	Personal-ization	1-way / 2-way	Adherence outcome assessment methods	Primary adherence outcome measure	Biochemical outcome assessment methods
Safren et al., 2003	every day at 9AM	General content	No	1-way	**MEMs cap** at 2 and 12 weeks: Continuous.	Proportion of medication taken as prescribed	-
ACTG 731 study team., 2008	each of weeks 1 to 12, 14, and 16	Medical content	Yes	2-way	**Self-report** (ACTG) at 4, 16, 32, 48, and 64 weeks: Continuous.	Proportion of medication taken as prescribed	-
Simoni et al., 2009	daily ≥3 pager messages in 2 months, gradually tapered in 3th month	Medical content	Yes	2-way	① Self-report (SMAQ) at 2 weeks and 3/6/9 months: Dichotomized at 100%. ②**MEMs cap** at 2 weeks and 3/6/9 months: Continuous.	Proportion of medication taken as prescribed	① HIV-1 RNA Viral Load (log10 copies/mL) at baseline and 3/6/9 months follow up: Continuous. ② CD4+ cell counting (cells/mm3) at baseline and 3/6/9 months follow up: Continuous.
WelTel Kenya1	Weekly (Monday morning)	General	No	2-way	**Self-report (pills taken)** at 6	Proportion of patients with	Viral load: Dichotomized at

study team., 2010		content			and 12 month: Dichotomized at 95%.	good adherence (take 95% medication or more)	virologically suppressed ＜ 400 copies/mL or virologic failures ≥400 copies/mL.
Pop-Eleches et al., 2011 (1)	daily SMS sent at 12 PM	General content	No	1-way	①**MEMs cap** during each 12-week period: Dichotomized at 90%.<br>②treatment interruptions lasting at least 48 h: Yes/Not.	Proportion of patients with good adherence (take 90% medication or more)	-
Pop-Eleches et al., 2011 (2)	weekly SMS sent at 12 PM	General content	No	1-way	①**MEMs cap** during each 12-week period: Dichotomized at 90%.<br>②treatment interruptions lasting at least 48 h: Yes/Not.	Proportion of patients with good adherence (take 90% medication or more)	-
CAMPS study team., 2012	Weekly (Wednesday 9AM)	General content	No	2-way	① **Self-report (VAS)**: Dichotomized at 95% at 3 and 6 month, Continuous at baseline.<br>②Self-report (no missed doses):	Proportion of medication taken as prescribed	CD4+ cell counting at Baseline, 3 months. (6 months insufficient data)<br>(insufficient data of all viral

					Dichotomized at 100%.<br>③ Pharmacy Refill record: Continuous.(Result cannot be meta-analyzed)		load)
da Costa et al., 2012	Weekends and alternate days during weekdays	General content	No	1-way	①Self-report adherence at each 4-month: Dichotomized at 95%.<br>②Pill counting at each 4-month: Dichotomized at 95%.<br>③**MEMs cap** at each 4-month: Dichotomized at 95%.	Proportion of patients with good adherence (take 95% medication or more)	-
Hersch et al., 2013	Daily medication schedule	Medical content	Yes	2-way	**MEMs Cap** at baseline, 3-, 6-, and 9-month: Continuous.	Proportion of medication taken as prescribed	-
HIVIND study team., 2014	once a week at a time selected by each patient	Medical content	No	2-way	**Pill counting** at weeks 4, 8, and 12, and then every 12 weeks until 96-week: Dichotomized at 95%;	Proportion of patients with good adherence (take 95% medication or more)	Viral load: Dichotomized at virologically suppressed ≤400 copies/mL or virologic failures ＞400 copies/mL.
ACTG 5031 study	1 to 3 days after starting	Medical	Yes	2-way	① **Self-report (ACTG II)** at	Proportion of medication	-

team., 2014	ART, and then on weeks 1, 2, 3, 6, 10, 14, 18, 22, 26, and every 8 weeks thereafter	content			weeks 4, 16, 32, 48, and then every 16 weeks thereafter: Continuous.<br>② Self-report (ACTG II) at weeks 4, 16, 32, 48, and then every 16 weeks thereafter: Dichotomized at 95%.	taken as prescribed	
Sabin et al., 2015	the device was not opened within 30 minutes of the scheduled dose time	General content	Yes	1-way	①EAMD (Wisepill) at month 3 and month 9: Dichotomized at 95%.<br>②**EAMD (Wisepill)** at month 3 and month 9: Continuous.	Proportion of medication taken as prescribed	① CD4+ cell counting at baseline and month 9.<br>② Viral load at baseline, 9-month follow up: Dichotomized at virologically suppressed ≤50 copies/mL or virologic failures ＞ 50 copies/mL.
Ingersoll et al., 2015	daily queries of medication dosing, mood twice daily,	General content	No	2-way	**Pharmacy refill record** at pre-intervention, post-intervention,	Proportion of medication taken as prescribed	-

	and substance use once daily				and 3-month follow-up: Continuous.		
Belzer et al., 2015	Monday through Friday (excluding holidays) for 24 weeks, once or twice a day	Medical content	No	2-way	①Self-report (VAS) at baseline, 24 weeks and 48 weeks follow up: Dichotomized at 90%.<br>② **Self-report (VAS)** at baseline, 24 weeks and 48 weeks follow up: Continuous;	Proportion of medication taken as prescribed	① Viral Load (log10 copies/mL) at baseline, 24 weeks and 48 weeks follow up: Continuous.<br>② Viral load at baseline, 24 weeks and 48 weeks follow up: Dichotomized at virologically suppressed ＜400 copies/mL or virologic failures ≥400 copies/mL.
Orrell et al., 2015	the device was not opened within 30 minutes of the scheduled dose time	General content	Yes	1-way	① **EAMD (Wisepill)**: Continuous.<br>② Proportion With 72 Hours Treatment Interruption: binary indicator(result cannot be meta-	Proportion of medication taken as prescribed	Viral load: Dichotomized at virologically suppressed ≤40 copies/mL or virologic failures ＞40 copies/mL.

					analyzed).		
Garofalo et al., 2016	daily 2 text reminders	General content	Yes	2-way	①Self-report (VAS) at baseline, 3 months and 6 months follow up: Dichotomized at 90%.<br>② **Self-report (VAS)** at baseline, 3 months and 6 months follow up: Continuous.	Proportion of medication taken as prescribed	① Viral Load (log10 copies/mL) at baseline, 3 months and 6 months follow up: Continuous.<br>②Viral load: Dichotomized at virologically suppressed ≤75 copies/mL or virologic failures ＞75 copies/mL.
Ruan et al., 2017	First 3 months: 5 text messages every Monday, Wednesday, Friday, and weekends.<br>Last 3 months: 3 text messages in every Monday, Wednesday, and Saturday.	Medical content	No	2-way	①**Self-report (VAS)** at baseline and 6 months follow-up: Continuous.<br>② Self-Report (CPCRA) at baseline and 6 months follow-up: Dichotomized at 100%.	Proportion of medication taken as prescribed	CD4+ cell counting at baseline and 6 months follow-up: Continuous.
Reid et al., 2017	SMS reminders three days	General	No	1-way	**Pharmacy refill record**:	Proportion of patients with	① Viral Load (log10

	prior, one day prior, and the morning of the scheduled monthly pharmacy pickup	content			Dichotomized at 100%.	good adherence (6 or more vists)	copies/mL) at baseline and at last visit in 6 months: Continuous.<br>②CD4+ cell counting at baseline and at last visit in 6 months: Continuous.
Abdulrahman et al., 2017	weekly SMS medication reminders at 9am every Monday, SMS reminder 3 days prior to scheduled clinic appointments, and an average of 90sec lunch hour telephone call reminders a day prior to scheduled clinic appointment	General content	No	1-way	① Self-report (AACTG) at baseline and 6 months follow up: Dichotomized at 95% (>95% adherence = missed <3 doses of durgs/month, ≤95% adherence = missed ≥3 doses of durgs/month);<br>② **Self-report (AACTG)** at baseline and 6 months follow up: Continuous;	Proportion of medication taken as prescribed	① CD4+ cell counting at baseline and 6 months follow up: Continuous.<br>② Viral Load (log10 copies/mL) at baseline and 6 months follow up: Continuous.
Linnemayr et al.,	Every Sunday at 9AM	General	No	1-way	①**MEMS Cap** at baseline and	Proportion of medication	-

2017 (1)		content			48 weeks follow up: Continuous.<br>②MEMS Cap at baseline and 48 weeks follow up: Dichotomized at 90%.<br>③treatment interruptions lasting at least 48 h: Yes/Not.	taken as prescribed	
Linnemayr et al., 2017 (2)	Every Sunday at 9AM	General content	No	2-way	①**MEMS Cap** at baseline and 48 weeks follow up: Continuous.<br>②MEMS Cap at baseline and 48 weeks follow up: Dichotomized at 90%.<br>③treatment interruptions lasting at least 48 h: Yes/Not.	Proportion of medication taken as prescribed	-
